# The Influence of Pasture and Non-pasture-Based Feeding Systems on the Aroma of Raw Bovine Milk

**DOI:** 10.3389/fnut.2022.841454

**Published:** 2022-03-10

**Authors:** Holly J. Clarke, Ellen Fitzpatrick, Deirdre Hennessy, Maurice G. O'Sullivan, Joseph P. Kerry, Kieran N. Kilcawley

**Affiliations:** ^1^Food Quality and Sensory Science, Teagasc Food Research Centre, Fermoy, Ireland; ^2^Sensory Group, School of Food and Nutritional Sciences, University College Cork, Cork, Ireland; ^3^Teagasc Animal and Grassland Research and Innovation Centre, Cork, Ireland; ^4^Food Packaging Group, School of Food and Nutritional Sciences, University College Cork, Cork, Ireland

**Keywords:** milk, pasture, aroma, olfactometry, bovine

## Abstract

Aroma-active compounds in raw bovine milk produced from cows fed perennial ryegrass (GRS) or total mixed ration (TMR) consisting of grass silage, maize silage, and concentrates were identified by direct immersion sorptive extraction (DI Hi-Sorb), coupled with gas-chromatography-mass spectrometry and olfactometry using odour intensity (OI) and aroma extraction dilution analysis (AEDA). Ninety-nine volatile organic compounds (VOC) were identified in these raw GRS and TMR milk samples; 33 of which were also present in the feed and rumen samples from these diets. Only the abundance of 13 VOC varied significantly based on diet. However, the odours of both raw milks were quite distinct as aroma perception is not influenced by abundance alone but also by the odour activity of each VOC. Approximately, 30% of the VOC influenced the aroma perception of these raw milks. This study clearly highlighted the significant impact of VOC transferring from the diet that influenced the aroma perception of both raw GRS and TMR milk. The aroma of the raw TMR milk was more complex than that of the raw GRS milk, and many of the key dietary-derived-odour-active VOC likely arose during the production of the TMR feed as most were either derived from Maillard reactions or impacted by heat. Seventeen of the 44 odour activities detected differed between both sample types. This study has clearly demonstrated the impact of diet on the aroma perception of raw bovine milk.

## Introduction

Previous studies have demonstrated a significant effect of the feeding system on the composition of raw milk and, particularly, its impact on the fatty acid content (1). It is also established that diet has a significant impact on the volatile profile of bovine milk (1–3), and it may even be possible to use volatile organic compounds (VOC) to authenticate pasture-based dairy products (4, 5). VOC in bovine milk consists of a range of different chemical classes, including aldehydes, ketones, lactones, esters, alcohols, acids, terpenes, furans, hydrocarbons, pyrazines, and phenolic and sulphur compounds (1, 5–7). However, their potential impact on sensory perception depends upon their relative concentration and odour activity. Previous studies have reported direct transfer of VOC from bovine feed to milk (4, 8), and that compounds such as phytochemicals in the feed may be metabolised in the rumen to more volatile odour-active compounds in the milk (7). Evaluating raw milk enables those VOC originating from the bovine-feeding system to be more easily assessed, as other VOC arising from milk heat treatment or formed during shelf life by microbial activity are not present. Information on the aroma perceptions and intensities of individual VOC in raw milk from different feeding systems may also prove important when selecting raw milk for future applications. For example, further processing to generate commodity dairy products may positively or negatively alter and/or exacerbate specific odours that may impact consumer preference.

Gas-chromatography-olfactometry (GC-O) is a very useful approach to identify odour-active VOC in food products (9). GC-O refers to the use of human assessors to detect aroma-active VOC extracted and separated using GC in tandem with mass spectrometry and/or flame ionisation detection. Friedrich and Acree et al. (10) reviewed GC-O studies on dairy products including those on raw and pasteurised bovine milk and highlighted the significance of esters in raw milk and the creation of other VOC during pasteurisation. At that time, only 2 studies had been undertaken (11, 12), and both used vacuum distillation as the extraction process. Solvent-assisted flavour evaporation (SAFE) was subsequently used to extract VOC in pasteurised bovine milk (13) from cows on two distinct diets (pasture and total mixed ration). These authors found 66 odour-active VOC in bovine milk, and that diet only influenced the abundance of these VOC rather than creating unique VOC, except for γ-12:2 lactone (γ-dodec-cis-6, cis-9-dienolactone), which was absent in the milk derived from a pasture diet. Other authors (14) have used headspace solid phase micro-extraction (HS-SPME) to identify aroma-active VOC in bovine milk. These authors found 75 odour-active VOC in bovine milk, but only found differences in abundances of individual VOC, not any distinct VOC associated with diet, despite the fact that the milks were selected as either good-quality milk or deemed to be tainted with a “feed” off-flavour as determined by certified expert sensory graders. A recent study of raw bovine milk (15) has also used HS-SPME, but only identified 9 aroma-active VOC, consisting of 7 acids, 1 aldehyde, and 1 ketone. Although HS-SPME is widely applied as a volatile extraction technique, it has some well-known limitations, particularly relating to the low volume of the sorbent phase, which can result in VOC competition and migration during the equilibration phase (16), and by a propensity to preferentially extract very volatile low-molecular-weight VOC (3). Although SAFE is a well-established extraction technique, it is time-consuming, requires solvents and complex glass apparatus (17), and has poor reproducibility (18).

One of the potential reasons why so few GC-O studies have been undertaken on either raw or heat-treated bovine milk may be due to its subtle flavour, making it difficult to discern aroma characteristics. Thus, it is imperative that VOC are concentrated sufficiently prior to separation by GC in order to be more easily perceived by olfactometry panellists. More green or environmentally friendly automated or semi-automated extraction techniques such as stir bar sorptive extraction (SBSE) are used to identify favour compounds due to their ease of use, good reproducibility, and high sorption capability (19). These appear to be more effective as direct immersive procedures rather than as headspace extraction procedures (20). A new version of sorptive extraction called high-capacity sorptive extraction (HiSorb) was successfully utilised by Faulkner et al. (6) to profile VOC from bovine milk produced by different diets and outperformed HS-SPME in a study by Cheng et al. (21) to profile VOC in whole milk powder. Thus, HiSorb as a direct immersion technique (DI-Hisorb) utilising polydimethylsiloxane (PDMS) as the sorbent appears ideally suited for GC-O analysis of bovine milk.

This study is the first to determine if any differences exist in relation to odour-active VOC in milk from different diets (pasture vs. non-pasture), using DI-HiSorb (with a PDMS sorbent phase) in tandem with GC-O and GC-MS.

## Materials and Methods

### Experimental Design and Milk Collection

Fifty-four spring-calving Friesian cows based at the Teagasc Moorepark dairy farm (Fermoy, Co. Cork, Ireland) were allocated to experimental feeding groups (*n* = 18), namely outdoors on perennial ryegrass (*Lolium perenne L.)*, indoors on total mixed ration, which comprised a mixture of grass silage, maize silage, and concentrates, or on pasture-mixed ration (50:50 pasture: TMR) whereby the cows were outdoors by day and indoors by night. The TMR diet consisted of, on a DM basis, 7.15 kg of grass silage, 7.15 kg of maize silage, and 8.3 kg of concentrates. Each cow received TMR *ad libitum*. The pasture-based cows consumed ~18 kg of dry matter per day measured by pre and postgrazing sward heights using the rising plate metre (Jenquip, Feilding, New Zealand), whereas pregrazing herbage mass was measured with an Etesia mower (Etesia UK Ltd., Warwick, UK). The full composition, including the chemical composition of the diets, was outlined by O'Callaghan et al. (1). A Latin Square design was employed whereby each group of 18 cows received either GRS, TMR, or pasture-mixed ration for 16 days and were then transferred to one of the other two diets for another 16 days, which resulted in each group of cows receiving each diet treatment over 48 days. Days 1–14 were used to acclimatise the cows to the feeding regimens, and samples were collected on Days 15 and 16. In this instance, because large amounts of milk were required for other processing, the morning milk from Day 14 was included in this study.

Only milk from the cows that received just the grass or just the total mixed ration diets were considered for this study and were denoted as GRS and TMR, respectively. Morning milk from the GRS and TMR diets was collected at 07:30 and stored at 4°C in designated 5,000-L refrigerated tanks until the evening milk (15:30) was added and the tank was stirred. Milk samples from five separate milkings over 3 days (3 mornings and 2 evenings) were taken and pooled together during the final 3 days of each 16-day feeding period. The samples were stored in sterile plastic containers at refrigeration temperature, prior to GC-MS and GC-O analysis, which occurred <24 h later.

#### Feed Sampling

Feed samples for GRS and TMR were taken from pasture paddocks on the corresponding milk collection day (Day 16). Pasture samples were cut just above the root from various sections across the paddocks. TMR samples were taken directly from the cow feeders. One sample was taken from each feeder (*n* = 18) and pooled together. The samples were frozen at −18°C until required for analysis. Sixty grammes of GRS and TMR samples were each blended with 150-ml dH_2_O until homogeneous and analysed immediately.

#### Rumen Sampling

Rumen samples were taken from cannulated cows (*n* = 3 per feeding system) in the morning and evening on Days 15 and 16 of the 16-day feeding period. The rumen fluid was separated from the solid portion *via* cheese cloth filtration and was denoted as RF. The solid portion was blended until homogenous and was denoted as RB. Samples were frozen at −18°C until required for analysis. Directly prior to analysis, the three-morning and three-evening RF and RB samples from Days 15 and 16 were pooled together for GRS (*n* = 12) and TMR (*n* = 12).

### Volatile Compound Analysis by HiSorb Gas-Chromatography Mass-Spectrometry

The extraction of compounds from the feed, rumen, and raw milk samples was carried out using conditioned (50°C for 10 min followed by 300°C for 30 min) HiSorb PDMS probes (Product Code: H1-AXAAC; Markes International Ltd., Bridgend, UK). Milk samples (10 ml), feed samples (15 g), and RF and RB samples (15 g) were placed in 20-ml crimp-top, round-bottomed clear vials (Product Code: C-VCC20; Markes International Ltd., Bridgend, UK) and capped with HiSorb-P1 crimp caps and HiSorb septa (Product Code: C-HSPCCS; Markes International Ltd., Bridgend, UK). NaCl (2.5 g) (Merck Ireland; Arklow, Co. Wicklow, Ireland) and 100-μl internal standard (4-methyl-2-pentanol; 500 μl of 1,000 ppm stock solution in 10-ml dH_2_O) were added to each milk sample prior to extraction. HiSorb probes were fully immersed in the feed, rumen, and milk samples for 1 h at 40°C with agitation at 400 rpm using a HiSorb agitator (Part No.: U-HSAG-20; Markes International Ltd., Bridgend, UK). After extraction, the probes were removed and rinsed with dH_2_O, and dried with lint free paper. The probes were placed inside empty thermal desorption (TD) tubes (Product Code: C0-AXXX-0000; Markes International Ltd., Bridgend, UK) and capped with brass storage caps fitted with one-piece PTFE ferrules (Product Code: C-CF020; Markes International Ltd., Bridgend, UK) until analysis. All extractions were carried out at the same time for each sample type. The brass caps were replaced by inert-coated stainless steel DiffLok caps (Product Code: C-DLS10; Markes International Ltd., Bridgend, UK) immediately prior to analysis. The GC-MS conditions for the HiSorb desorption analysis were performed as described by Vilar et al. (22). All samples were analysed in triplicate. The system cheque standards used were 1-butanol, dimethyl disulfide, butyl acetate, cyclohexanone, benzaldehyde, and 2-phenyl-D5-ethanol.

### Gas-Chromatography Olfactometry Odour Intensity Analysis

The DI-HiSorb extractions of the raw milks for GC-O evaluation were carried out as described in Section Volatile Compound Analysis by HiSorb Gas-Chromatography Mass-Spectrometry. Desorption of the HiSorb probes was automated by a Markes Centri system (Markes International Ltd., Bridgend, UK). Probes were desorbed for 10 min at 280°C onto the material emissions cold trap (Part No: U-T12ME-2S), which was held at 30°C. Prior to desorption of the trap, a 1-min pre-purge step of nitrogen gas was carried out with a 1:50 split. Trap desorption was performed by heating the trap to 300°C and holding it for 5 min. GC conditions were performed as described by Vilar et al. (23). All the samples were analysed in a splitless mode and were evaluated by each panellist (*n* = 5) in duplicate.

#### GC-O Analytical Standards

Olfactory training standards were of analytical grade; ethyl butyrate, octanal, p-cresol, and dimethyl disulphide and heptanal of ≥ 99% and ≥ 95% purity, respectively (Merck Ireland, Arklow, Co. Wicklow, Ireland), were prepared at 0.3% (w/v) in methanol and stored at −18°C until required. For each GC-O training session, a stock solution was diluted to 0.03% (w/v) in distilled water to allow the odours to be of adequate potency.

Five experienced sensorial assessors evaluated the odour perceptions of the VOC in the raw GRS and TMR milks. Sniffing time was approximately 29 min, and each assessor carried out one session per day. Prior to sample analysis, the panellists were exposed to a standard stock solution (as described in Section GC-O Analytical Standards), designed for GC-O training, comprised of 5 compounds: dimethyl disulphide (“sulphur,” “decomposing”), ethyl butyrate (“fruity,” “pineapple”), heptanal (“fatty,” “green”), octanal (“orange,” “fruity”), and ρ-cresol (“barnyard”). This step allowed panellists to familiarise themselves with the GC-O process and software, as well as the range of odours they could potentially encounter during the GC-O analysis of these raw milk samples. Panellists did not receive formal training on all compounds identified in raw milk as it was deemed impractical due to time constraints. Similar to the study by Vilar et al. (23), the panellists were asked to rate (i) the intensity of the eluted aroma using a four-point category scale (1 = weak, hardly recognisable odour; 2 = clear but not intense odour; 3 = intense odour; 4 = very intense odour), recorded by a Gerstel OID Interface/ODP-Recorder (Anatune Ltd, Cambridge, UK), and (ii) the odour perceived by voice recording. Significant odourants were those that were perceived by at least three of the five assessors. Compound identifications were carried out as described by Vilar et al. (23). Odour intensities (OI) for each compound were determined by averaging the panellists' intensity ratings; thus, all values have an OI range between 1 and 4.

### Gas-Chromatography Oflactometry Aroma Extraction Dilution Analysis (AEDA)

Aroma extraction dilution analysis was carried out on the GRS and TMR milks as described by Garvey et al. (24). Briefly, the technique was carried out by manipulation of the desorption split ratio (25). The split ratio was adjusted to 1:1, 1:2, 1:5, 1:10, 1:20, 1:50, 1:100, and 1:150, allowing for adequate dilution to determine the most odour-active compounds. Undertaking AEDA using the split approach removed any potential matrix effects that could occur if the sample was diluted. The assessor who demonstrated the highest olfactory perception in the previous analysis was chosen for the AEDA study. The last split ratio at which a compound could be perceived was referred to as the factor dilution (FD) for that compound. AEDA analysis was carried out in duplicate for each sample.

### Statistical Analysis

Statistical analysis relating to the volatile compounds identified in raw milk samples was carried out using the independent samples *t*-test in Statistical Package for the Social Sciences (SPSS) (IBM corp., Armonk, NY, USA). Principal component analysis biplots of the volatile and odour descriptor data were constructed using the factoextra and FactoMinoR packages within R [v. 3.4.1; (26)]. All sensory and volatile data were averaged before analysis. **Figure 2** was created using MetaboAnalyst v. 5.0.

## Results and Discussion

The VOC identified in raw GRS and TMR bovine milk are provided in Table 1. The volatile compounds identified in the feed, RF, RB, and raw milk samples by DI-HiSorb GC-MS are outlined in Supplementary Table 1. Thirty-three VOC, consisting of 5 acids, 9 alcohols, 6 aldehydes, 1 furan, 5 hydrocarbons, 4 ketones, 1 lactone, 1 pyridine, and 1 other, were present across all sample types (feed, RB, RF, and raw milk; Table 2).

**Table 1:** Volatile compounds identified in raw bovine milk from cows-fed grass (GRS) or total mixed ration (TMR) by HiSorb-GC-MS.

**Compound**	**CAS no**	**RI**	**IM**	**GRS**	**TMR**	***p*** **-value**
**Acids**						
Formic acid	64-18-6	605.8	MS	8.53 × 10^4^	6.58 × 10^4^	NS
Acetic acid	64-19-7	662.8	MS, IHL, LRI	1.96 × 10^6^	2.57 × 10^6^	NS
Propanoic acid	79-09-4	786	MS, IHL, LRI	2.49 × 10^5^	2.64 × 10^5^	NS
2-Methylpropanoic acid	79-31-2	841.8	MS, IHL, LRI	1.92 × 10^4^	1.44 × 10^4^	NS
2-Methyl-2-propenoic acid	79-41-4	882.2	MS	3.78 × 10^4^	5.37 × 10^4^	NS
Butanoic acid	107-92-6	882.5	MS, IHL	7.93 × 10^5^	1.10 × 10^6^	NS
3-Methylbutanoic acid	503-74-2	932.8	MS, IHL	ND	4.35 × 10^4^	NS
2-Methylbutanoic acid	116-53-0	937.1	MS, IHL	2.07 × 10^4^	1.13 × 10^5^	^*^
1-Methylpropyl ester butanoic acid	819-97-6	961.9	MS	6.56 × 10^3^	ND	NS
Pentanoic acid	109-52-4	973.9	MS	1.60 × 10^5^	3.77 × 10^5^	NS
Hexanoic acid	142-62-1	1069.7	MS, IHL, LRI	7.81 × 10^5^	1.67 × 10^6^	^*^
Heptanoic acid	111-14-8	1164.1	MS, IHL	1.82 × 10^5^	3.53 × 10^5^	NS
Octanoic acid	124-07-2	1261.9	MS	1.41 × 10^6^	3.45 × 10^6^	^*^
Benzoic acid	65-85-0	1285.2	MS	2.35 × 10^5^	3.40 × 10^4^	NS
Nonanoic acid	112-05-0	1353.8	MS, IHL	3.98 × 10^5^	6.88 × 10^5^	NS
Decanoic acid	334-48-5	1452.3	MS, IHL	2.97 × 10^6^	6.71 × 10^6^	^*^
Hydrocinnamic acid	501-52-0	1460	MS	8.27 × 10^4^	2.89 × 10^4^	NS
Undecanoic acid	112-53-8	1544	MS, LRI	8.26 × 10^4^	1.45 × 10^5^	NS
Dodecanoic acid	143-07-7	1640.2	MS	2.02 × 10^6^	1.94 × 10^6^	NS
Tetradecanoic acid	544-63-8	1839.7	MS	4.39 × 10^5^	2.91 × 10^5^	NS
**Alcohols**						
Ethanol	64-17-5	504.3	MS, IHL, LRI	3.51 × 10^6^	2.74 × 10^6^	NS
1-Butanol	71-36-3	688.9	MS, IHL	9.70 × 10^3^	ND	NS
3-Methylbutanol	123-51-3	774.5	MS, IHL	2.20 × 10^5^	1.19 × 10^4^	NS
4-Methyl-2-pentanol	108-11-2	796.4	MS, IHL, LRI	7.85 × 10^3^	3.54 × 10^3^	NS
1-Pentanol	71-41-0	810.6	MS, IHL, LRI	1.07 × 10^5^	1.95 × 10^4^	^*^
3-Furanmethanol	4412-91-3	868.3	MS	1.81 × 10^5^	2.33 × 10^5^	NS
1-Hexanol	111-27-3	917.2	MS, IHL, LRI	1.88 × 10^4^	3.75 × 10^3^	NS
2-Furanmethanol	98-00-0	929.5	MS, LRI	5.21 × 10^6^	6.80 × 10^6^	NS
2-Butoxyethanol	111-76-2	952	MS, IHL, LRI	1.87 × 10^4^	2.38 × 10^3^	NS
3-Methyl-1-hexyn-3-ol	4339-05-3	1046.4	MS	9.68 × 10^2^	6.44 × 10^2^	NS
Dihydroxyacetone	96-26-4	1046.5	MS	2.76 × 10^4^	1.35 × 10^4^	NS
2-Ethylhexanol	104-76-7	1078.1	MS, IHL, LRI	9.46 × 10^4^	7.65 × 10^4^	NS
Phenylethyl Alcohol	60-12-8	1193	MS, IHL, LRI	1.09 × 10^4^	1.42 × 10^4^	NS
1-Octanol	111-87-5	1120.1	MS, IHL	7.39 × 10^4^	5.37 × 10^4^	NS
2-Phenoxyethanol	122-99-6	1320.4	MS, IHL, LRI	2.94 × 10^5^	1.55 × 10^5^	NS
1-Dodecanol	112-53-8	1523.3	MS, LRI	1.35 × 10^5^	1.05 × 10^5^	NS
Tetradecanol	112-72-1	1724.2	MS	1.49 × 10^5^	1.78 × 10^5^	NS
**Aldehydes**						
Acetaldehyde	75-07-0	449.5	MS, IHL, LRI	5.50 × 10^6^	4.97 × 10^6^	NS
2-Methylpropanal	78-84-2	582.5	MS, IHL, LRI	ND	4.93 × 10^5^	^*^
3-Methylbutanal	590-86-3	650.4	MS, IHL, LRI	2.43 × 10^4^	2.24 × 10^4^	NS
Hexanal	66-25-1	828.9	MS, IHL	2.21 × 10^5^	1.85 × 10^5^	NS
Furfural	98-01-1	891.7	MS, IHL, LRI	1.09 × 10^6^	1.31 × 10^6^	NS
(E)-2-Hexenal	6728-26-3	901.2	MS	1.35 × 10^4^	5.05 × 10^3^	NS
Heptanal	111-71-7	938.7	MS, IHL, LRI	2.13 × 10^5^	1.60 × 10^5^	NS
Benzaldehyde	100-52-7	1019.3	MS, IHL	2.19 × 10^5^	2.03 × 10^5^	NS
5-methyl furfural	620-02-0	1032.2	MS	1.73 × 10^5^	2.07 × 10^5^	NS
Octanal	124-13-0	1043.6	MS, IHL	3.07 × 10^5^	2.27 × 10^5^	NS
(E,E)-2,4-Heptadienal	4313-03-5	1074.2	MS, LRI	2.56 × 10^4^	1.39 × 10^3^	^*^
Benzeneacetaldehyde	122-78-1	1108.5	MS, IHL, LRI	2.76 × 10^4^	3.94 × 10^4^	NS
Nonanal	124-19-6	1147.6	MS, IHL, LRI	1.11 × 10^6^	7.50 × 10^5^	NS
Decanal	112-31-2	1252.6	MS, IHL, LRI	4.16 × 10^5^	3.02 × 10^5^	NS
Dodecanal	112-54-9	1457.2	MS, IHL	5.68 × 10^4^	ND	NS
Tridecanal	10486-19-8	1558.7	MS, LRI	2.28 × 10^4^	8.22 × 10^3^	NS
**Esters and Ethers**						
Butyl acetate	123-86-4	834.2	MS, IHL, LRI	7.36 × 10^3^	3.51 × 10^3^	NS
Ethyl pentanoate	539-82-2	923.3	MS, IHL, LRI	1.05 × 10^5^	5.34 × 10^3^	NS
Butyl butanoate	100-52-7	1021.4	MS, IHL, LRI	8.17 × 10^3^	ND	NS
Ethyl hexanoate	123-66-0	1024.9	MS, IHL, LRI	1.28 × 10^5^	1.41 × 10^4^	NS
Dimethyl succinate	106-65-0	1085.3	MS	2.49 × 10^3^	1.26 × 10^3^	NS
Methyl-2-furoate	611-13-2	1170	MS	4.18 × 10^5^	4.88 × 10^5^	NS
Ethyl octanoate	106-32-1	1225.7	MS, IHL, LRI	8.64 × 10^4^	2.02 × 10^4^	NS
Ethyl decanoate	110-38-3	1422.5	MS, IHL, LRI	9.29 × 10^4^	3.18 × 10^4^	NS
Ethyl dodecanoate	106-33-2	1620	MS, IHL, LRI	1.13 × 10^4^	7.60 × 10^3^	NS
**Furans**						
2-Methylfuran	79-09-4	793.9	MS	1.36 × 10^4^	2.25 × 10^4^	NS
2-Pentylfuran	3777-69-3	1008.9	MS, IHL, LRI	7.76 × 10^3^	1.31 × 10^4^	NS
Isomaltol	3420-59-5	1040	MS, LRI	1.22 × 10^6^	1.10 × 10^6^	NS
**Hydrocarbons and Benzenes**						
Toluene	108-88-3	773.5	MS, IHL	1.14 × 10^5^	1.53 × 10^4^	^*^
p-Xylene	106-42-3	888.2	MS, IHL, LRI	2.25 × 10^4^	1.16 × 10^4^	NS
Phenol	108-95-2	1104.5	MS, IHL, LRI	1.00 × 10^5^	8.47 × 10^4^	NS
p-Cresol	106-44-5	1195	MS, IHL, LRI	5.82 × 10^5^	2.90 × 10^5^	NS
Benzothiazole	95-16-9	1296.2	MS, IHL, LRI	7.36 × 10^4^	3.39 × 10^4^	^*^
2-Methoxy-4-vinylphenol	7786-61-0	1411.9	MS, IHL	3.63 × 10^5^	4.19 × 10^4^	NS
Indole	110-38-3	1430.8	MS, IHL, LRI	1.49 × 10^4^	1.61 × 10^4^	NS
**Ketones**						
Acetone	67-64-1	491.9	MS, IHL, LRI	2.40 × 10^5^	3.94 × 10^5^	NS
2,3-Butanedione (Diacetyl)	431-03-8	574.6	MS, IHL, LRI	1.12 × 10^5^	1.65 × 10^5^	NS
2-Pentanone	107-87-9	704.2	MS, IHL, LRI	4.38 × 10^4^	3.61 × 10^4^	NS
1-Hydroxy-2-propanone	116-09-6	709.8	MS, IHL	6.83 × 10^5^	9.41 × 10^5^	NS
Methyl Isobutyl Ketone	108-10-1	764.5	MS, IHL	2.34 × 10^4^	1.34 × 10^4^	NS
2-Heptanone	110-43-0	931.4	MS, IHL, LRI	1.37 × 10^5^	1.97 × 10^4^	NS
Dihydroxyacetone	96-26-4	1046.5	MS	2.76 × 10^4^	1.35 × 10^4^	NS
Acetophenone	98-86-2	1132.5	MS, IHL, LRI	6.74 × 10^4^	5.18 × 10^4^	NS
2-Undecanone	112-12-9	1343.3	MS, IHL	1.02 × 10^5^	7.08 × 10^4^	NS
2-Tridecanone	593-08-8	1546.2	MS	2.60 × 10^5^	1.79 × 10^5^	NS
**Lactones**						
γ-Butyrolactone	96-48-0	1021.1	MS, IHL, LRI	2.85 × 10^4^	6.91 × 10^4^	NS
2(5H)-Furanone	497-23-4	1026.3	MS, LRI	7.72 × 10^5^	1.08 × 10^6^	NS
γ-Hexalactone	695-06-7	1163	MS, IHL, LRI	1.95 × 10^4^	8.67 × 10^4^	NS
2(3H)-Furanone, dihydro-4-hydroxy-	5469-16-9	1382	MS	1.43 × 10^6^	1.71 × 10^6^	NS
γ-Nonalactone	104-61-0	1485	MS, IHL, LRI	3.34 × 10^4^	1.84 × 10^5^	^*^
**Pyrazines and Pyridines**						
Pyrazine	290-37-9	753	MS	1.59 × 10^4^	2.86 × 10^4^	^*^
Pyridine	110-86-1	775.8	MS, IHL	6.22 × 10^3^	6.74 × 10^3^	NS
2,5-Dimethylpyrazine	123-32-0	950	MS, IHL, LRI	ND	7.27 × 10^4^	NS
2,3-Dimethylpyrazine	5910-89-4	959	MS, IHL, LRI	3.77 × 10^3^	5.71 × 10^4^	NS
**Sulphurs**						
Methanethiol	90500-11-1	460.1	MS, IHL, LRI	4.55 × 10^5^	4.79 × 10^5^	NS
Dimethyl disulfide	624-92-0	754.6	MS	3.74 × 10^4^	7.44 × 10^4^	NS
Dimethyl sulfone	67-71-0	1056	MS, IHL, LRI	2.49 × 10^3^	2.08 × 10^4^	^*^
**Other**						
2-Methyl-1H-pyrrole	636-41-9	918.1	MS, IHL, LRI	4.94 × 10^3^	3.94 × 10^3^	NS
3-Methyl-2,5-furandione	110-00-9	1050.8	MS	4.96 × 10^5^	8.05 × 10^5^	NS
1H-Pyrrole-2,5-dione	541-59-3	1102.9	MS	8.09 × 10^3^	9.32 × 10^3^	NS
Maltol	118-71-8	1193	MS, LRI	1.28 × 10^6^	2.39 × 10^6^	^*^
2-Pyrrolidone	88-12-0	1196	MS	2.28 × 10^5^	9.37 × 10^4^	NS

*Levels of volatile compounds are expressed as abundances [mean values from 3 extractions from each raw milk sample (GRS and TMR)]*.

*LRI: retention index on a DB-624 UI column; IM: identification method; MS: spectra comparison using NIST mass spectral database; IHL: in-house library created using authentic compounds with target and qualifier ions and linear RI for each compound; LRI: RI agree with literature values*.

*ND, not detected; NS, not significant*.

* *p < 0.05; the significance of raw milk samples based on diet according to the Independent Samples t-test*.

**Table 2:** Volatile organic compounds present in all samples [feed (grass (GRS) and TMR, rumen fluid (RF), rumen blended (RB), raw grass (GRS), and TMR milk].

**Number**	**Compound**	**CAS no**.	**LRI^**a**^**	**Grass feed**	**TMR feed**	**Grass RF**	**TMR RF**	**Grass RB**	**TMR RB**	**Raw GRS milk**	**Raw TMR milk**	**Occurrence**
**Acids**												
1	Acetic acid	64-19-7	662.8	3.72 × 10^6^	2.29 × 10^7^	2.61 × 10^7^	4.58 × 10^7^	7.11 × 10^7^	5.26 × 10^7^	1.96 × 10^6^	2.57 × 10^6^	AO
2	Propanoic acid	79-09-4	786	1.69 × 10^6^	1.16 × 10^7^	1.19 × 10^7^	2.72 × 10^7^	3.16 × 10^7^	2.70 × 10^7^	2.49 × 10^5^	2.64 × 10^5^	AO
3	Butanoic acid	107-92-6	882.5	3.68 × 10^6^	9.57 × 10^7^	3.03 × 10^7^	8.33 × 10^7^	1.03 × 10^8^	1.79 × 10^8^	7.93 × 10^5^	1.10 × 10^6^	AO
4	Dodecanoic acid	143-07-7	1,640.2	6.15 × 10^5^	7.63 × 10^6^	3.36 × 10^6^	1.35 × 10^7^	2.39 × 10^6^	5.12 × 10^6^	2.02 × 10^6^	1.94 × 10^6^	AO
5	Hexanoic acid	142-62-1	1,069.7	1.01 × 10^6^	8.67 × 10^7^	9.27 × 10^6^	1.14 × 10^8^	2.05 × 10^7^	1.57 × 10^8^	7.81 × 10^5^	1.67 × 10^6^	A
**Alcohols**												
6	Ethanol	64-17-5	504.3	8.61 × 10^5^	5.18 × 10^6^	3.79 × 10^6^	3.52 × 10^6^	2.21 × 10^6^	3.30 × 10^6^	3.51 × 10^6^	2.74 × 10^6^	A
7	1-Butanol	71-36-3	688.9	1.10 × 10^5^	5.37 × 10^6^	2.40 × 10^6^	5.90 × 10^6^	5.69 × 10^6^	9.15 × 10^6^	9.70 × 10^3^	ND	A
8	1-Pentanol	71-41-0	810.6	9.06 × 10^5^	1.53 × 10^7^	2.16 × 10^6^	6.53 × 10^6^	2.98 × 10^6^	8.94 × 10^6^	1.07 × 10^5^	1.95 × 10^4^	AO
9	1-Hexanol	111-27-3	917.2	7.57 × 10^6^	7.47 × 10^6^	2.44 × 10^6^	1.95 × 10^7^	1.52 × 10^6^	1.93 × 10^7^	1.88 × 10^4^	3.75 × 10^3^	A
10	2-Ethylhexanol	104-76-7	1,078.1	1.61 × 10^6^	4.84 × 10^5^	1.40 × 10^6^	7.40 × 10^5^	2.19 × 10^5^	2.62 × 10^5^	9.46 × 10^4^	7.65 × 10^4^	AO
11	1-Octanol	111-87-5	1,120.1	2.44 × 10^5^	2.85 × 10^6^	1.14 × 10^5^	2.72 × 10^5^	1.92 × 10^5^	9.23 × 10^5^	7.39 × 10^4^	5.37 × 10^4^	AO
12	Phenylethyl Alcohol	60-12-8	1,193	1.31 × 10^6^	2.76 × 10^7^	2.92 × 10^6^	6.44 × 10^6^	1.80 × 10^6^	6.88 × 10^6^	1.09 × 10^4^	1.42 × 10^4^	AO
13	1-Dodecanol	112-53-8	1,523.3	2.69 × 10^5^	1.02 × 10^5^	8.39 × 10^5^	1.44 × 10^6^	1.99 × 10^5^	4.43 × 10^5^	1.35 × 10^5^	1.05 × 10^5^	A
14	Tetradecanol	112-72-1	1,724.2	2.36 × 10^4^	ND	3.60 × 10^4^	6.73 × 10^4^	ND	ND	1.49 × 10^5^	1.78 × 10^5^	AO
**Aldehydes**												
15	Acetaldehyde	75-07-0	449.5	1.06 × 10^6^	1.63 × 10^6^	2.97 × 10^6^	2.94 × 10^6^	3.30 × 10^6^	2.75 × 10^6^	5.50 × 10^6^	4.97 × 10^6^	A
16	3-Methylbutanal	590-86-3	650.4	1.15 × 10^6^	8.85 × 10^5^	2.16 × 10^5^	2.27 × 10^5^	5.16 × 10^5^	3.43 × 10^5^	2.43 × 10^4^	2.24 × 10^4^	A
17	Benzaldehyde	100-52-7	1,019.3	2.02 × 10^6^	3.67 × 10^6^	3.84 × 10^6^	3.11 × 10^6^	2.10 × 10^6^	1.66 × 10^6^	2.19 × 10^5^	2.03 × 10^5^	AO
18	Heptanal	111-71-7	938.7	8.52 × 10^5^	7.74 × 10^5^	6.81 × 10^5^	7.17 × 10^5^	3.01 × 10^5^	4.02 × 10^5^	2.13 × 10^5^	1.60 × 10^5^	AO
19	Decanal	112-31-2	1,252.6	1.12 × 10^6^	9.88 × 10^5^	1.41 × 10^6^	1.12 × 10^6^	6.27 × 10^5^	5.75 × 10^5^	4.16 × 10^5^	3.02 × 10^5^	AO
20	Octanal	124-13-0	1,043.6	5.92 × 10^5^	7.53 × 10^5^	9.14 × 10^5^	4.52 × 10^5^	3.06 × 10^5^	4.69 × 10^5^	3.07 × 10^5^	2.27 × 10^5^	A
**Furan**												
21	2-Pentylfuran	3777-69-3	1,008.9	4.94 × 10^5^	1.25 × 10^6^	1.35 × 10^6^	1.25 × 10^6^	4.47 × 10^5^	5.72 × 10^5^	7.76 × 10^3^	1.31 × 10^4^	AO
**Hydrocarbons and Benzenes**												
22	Toluene	108-88-3	773.5	5.71 × 10^5^	5.06 × 10^5^	3.20 × 10^7^	1.97 × 10^6^	7.03 × 10^6^	1.04 × 10^6^	1.14 × 10^5^	1.53 × 10^4^	AO
23	p-Xylene	106-42-3	888.2	7.52 × 10^5^	1.13 × 10^5^	6.36 × 10^5^	1.61 × 10^5^	2.36 × 10^5^	3.56 × 10^5^	2.25 × 10^4^	1.16 × 10^4^	A
24	Phenol	108-95-2	1,104.5	5.95 × 10^5^	1.85 × 10^6^	3.23 × 10^6^	5.08 × 10^6^	4.86 × 10^6^	6.17 × 10^6^	1.00 × 10^5^	8.47 × 10^4^	A
25	p-Cresol	106-44-5	1,195	9.80 × 10^5^	1.51 × 10^6^	2.25 × 10^8^	1.93 × 10^8^	2.06 × 10^8^	1.93 × 10^8^	5.82 × 10^5^	2.90 × 10^5^	AO
26	2-Methoxy-4-vinylphenol	7786-61-0	1,411.9	1.01 × 10^5^	1.16 × 10^7^	3.99 × 10^5^	3.14 × 10^5^	1.27 × 10^6^	5.93 × 10^5^	3.63 × 10^5^	4.19 × 10^4^	AO
**Ketones**												
27	Acetone	67-64-1	491.9	9.47 × 10^5^	1.81 × 10^6^	4.67 × 10^6^	2.72 × 10^6^	2.79 × 10^6^	1.87 × 10^6^	2.40 × 10^5^	3.94 × 10^5^	A
28	2,3-Butanedione	431-03-8	574.6	3.03 × 10^5^	1.08 × 10^6^	1.46 × 10^5^	4.79 × 10^5^	4.95 × 10^5^	6.61 × 10^5^	1.12 × 10^5^	1.65 × 10^5^	AO
29	2-Pentanone	116-09-6	704.2	5.74 × 10^4^	4.55 × 10^5^	1.24 × 10^5^	1.63 × 10^5^	6.37 × 10^5^	2.06 × 10^5^	4.38 × 10^4^	3.61 × 10^4^	A
30	2-Heptanone	110-43-0	931.4	3.27 × 10^5^	5.94 × 10^5^	9.14 × 10^5^	1.64 × 10^6^	1.53 × 10^5^	3.66 × 10^5^	1.37 × 10^5^	1.97 × 10^4^	AO
**Lactones**												
31	γ -Nonalactone	104-61-0	1,485	2.68 × 10^5^	2.16 × 10^7^	1.50 × 10^7^	1.20 × 10^7^	5.81 × 10^6^	6.84 × 10^6^	3.34 × 10^4^	1.84 × 10^5^	AO
**Pyrazines and Pyridines**												
32	Pyridine	110-86-1	775.8	1.04 × 10^4^	5.52 × 10^4^	6.43 × 10^4^	1.28 × 10^5^	1.29 × 10^4^	6.90 × 10^4^	6.22 × 10^3^	6.74 × 10^3^	A
**Other**												
33	2-Methyl-1H-pyrrole	636-41-9	918.1	9.44 × 10^3^	3.71 × 10^4^	3.14 × 10^4^	5.40 × 10^4^	1.70 × 10^5^	6.99 × 10^4^	4.94 × 10^3^	3.94 × 10^3^	A

*A, a compound identified in all sample types; O, a compound is odour active. Levels of volatile compounds are expressed as abundances (mean values from 3 extractions from each sample)*.

a *Retention index (RI) calculated from thermal desorption (TD) results on a DB-624 UI column*.

### Volatiles Identified in raw GRS and TMR Milk

Ninety-nine VOC were identified in raw milk by DI-HiSorb-GC-MS, which is significantly more than previous studies (10–15) and highlights the capability of the DI-HiSorb extraction technique (Table 1). Thirteen VOC varied significantly (*p* = 0.05) based on diet. Four of these VOC (1-pentanol, (E,E)-2,4-heptadienal, toluene, and benzothiazole) were significantly higher in raw GRS milk, and 10 [2-methyl butanoic acid, hexanoic acid, octanoic acid, decanoic acid, 2-methylpropanal, 3-/4-ethylphenol (tentative identification), γ-nonalactone, pyrazine, dimethyl sulfone, and maltol] were significantly higher in raw TMR milk. Only 1-methylpropyl ester butanoic acid, 1-butanol, dodecanal, and butyl butanoate were identified in raw GRS milk and not in raw TMR milk. Only 3-methylbutanoic acid, 2-methylpropanal, and 2,5-dimethylpyrazine were identified in raw TMR milk and not in raw GRS milk. These results generally concur with previous studies, which highlight no major differences in individual VOC but show significant differences in abundances due to diet (13, 14, 27, 28).

### Most Abundant Volatiles in Raw GRS Milk

The VOC that were statistically more abundant in the raw GRS milk diet consisted mainly of products of lipid oxidation [1-pentanoland (E,E)-2,4-heptadienal], metabolism of β-carotene (toluene), and/or derived from Maillard reactions (benzothiazole). Comparable to other studies, 1-pentanol and toluene were significantly higher in milk produced from cows-fed pasture (5, 6, 27). 1-Pentanol is a major product of lipid oxidation and likely relates to differences in the abundance of specific unsaturated fatty acids due to the different bovine diets (4). However, it is also likely that it was directly transferred from the diet, as it was present in all feed and rumen samples (Supplementary Table 1). Toluene was present in all feed and rumen samples with the greatest abundances in GRS RF (Supplementary Table 1). Toluene is not particularly odour active but has been suggested as a potential biomarker for pasture-fed bovine milk and associated dairy products, as it is a product of β-carotene metabolism in the rumen (4, 6). (E,E)-2,4-Heptadienal is another product of lipid oxidation, and was significantly higher in raw GRS milk and also likely related to higher levels of linolenic and linoleic acid due to the GRS diet, and is known to be quite an odour active (29). However, few studies have previously identified these VOC in milk. Benzothiazole was significantly higher in raw GRS milk and was present in only GRS feed and not in any rumen samples (Supplementary Table 1). Benzothiazole is thought to originate from phenylalanine through Maillard reactions (30) and has been described as having a burning rubber smell in milk; the odour of which increases with increasing heat treatment (11, 31, 32). Benzothiazole has also previously been found to have a greater aroma impact in milk derived from pasture than TMR and has a medium-odour threshold (1, 13).

#### Most Abundant Volatiles in raw TMR Milk

The range of VOC present at statistically (*p* = 0.05) higher abundances in the raw TMR milk encompassed numerous chemical classes and reflects a more complex VOC profile than in raw GRS milk. Both 2-methylbutanoic acid (a branched-chain fatty acid) and 2-methylpropanal (a branched-chain aldehyde) are products of Strecker degradation from isoleucine and leucine, respectively. 2-Methylpropanal has a characteristic musty aroma and a very low-odour threshold (13), and 2-methylbutanoic acid has a fruity, sweaty, rancid, burnt, sour aroma (14). A previous study identified 2-methylbutanoic acid in TMR feed but not in pasture feed (GRS or CLV); however, it was not detected in milk derived from these feeding systems (4). 2-Methylpropanal was absent in raw GRS milk but present at a relatively high abundance in raw TMR milk (Supplementary Table 1). These results generally contradict other studies, which have found that herbage-based diets tend to result in more products of amino acid metabolism in the resultant milk, as they typically have a high protein to digestible carbohydrate ratio than a concentrate diet, such as TMR (28, 33). However, it appears that 2-methylpropanal is also directly transferred from the diet as it was present in each feed and rumen sample (Supplementary Table 1). Dimethyl sulfone is derived from the oxidation of dimethyl sulphide, which may be formed *via* the metabolism of methionine and/or cysteine (27), or from heat-induced oxidation of methionine (34). It may also be transferred directly from plant-based diets (2), but this was not evident in this study (Supplementary Table 1). Similar to the branched acids and aldehydes, numerous studies have found higher abundances of dimethyl sulfone in bovine milk derived from a pasture-feeding system than from a concentrate-feeding system (2, 4, 28, 35), suggesting it is likely related to the increased availability of more digestible protein (methionine) in the pasture-based feeding system, which, again, contradicts the findings of this study. Faulkner et al. (6) found lower abundances of dimethyl sulfone in milk after pasteurisation, yet Moio et al. (11) only found a reduction in the abundance of dimethyl sulfone after UHT treatment. Therefore, heat treatment of milk or volatile extraction conditions appear to also influence its abundance. Vazquez-Landaverde et al. (34) suggested that, as the odour threshold of dimethyl sulfone is quite high, it is, therefore, unlikely to be a key aroma-active compound. Its odour had been described as sulphurous, hot milk, burnt, leather, and sweat like (11, 34).

The short chain fatty acids, hexanoic, octanoic, and decanoic were statistically (*p* = 0.05) higher in TMR milk and have the following aromas: hexanoic acid (unpleasant, chemical, caramel like), octanoic acid (intense, burnt milk or pudding), and decanoic acid (burnt, persistent, phenolic) (14). Short chain fatty acids are primarily produced by *de novo* synthesis in the mammary gland, which is impacted by diet but can also be directly transferred from the diet in free form (1). The evidence in this study also highlights the potential for direct transfer, as each of these acids was found in each feed and rumen sample (Supplementary Table 1). Each of these acids was also in greatest abundance in TMR feed and TMR RF. These acids are also produced by lipolytic activity from lipoprotein lipase or by esterases from psychrotrophic bacteria (36); however, as the milks in this study were treated in the same manner, it is more likely that the differences in short chain fatty acids were either directly or indirectly a result of diet. However, these results are in conflict with previous studies, which found no dietary impact on the abundance of free short chain fatty acids or that more free short chain fatty acids were associated with pasture feeding (6, 27, 31).

Pyrazines can be formed *via* the Strecker reaction driven by heat treatment (35) *via* Maillard browning or microbial metabolism (37). Clarke et al. (5) did not find any statistical difference in pyrazine between different bovine diets [GRS, GRS/Clover (CLV), and TMR] but did find statistical differences in the abundance of other pyrazines related to those diets (2,3,5-trimethyl-6-ethylpyrazine, 2,3-dimethyl-pyrazine, 3-ethyl-2,5-dimethyl-pyrazine, and trimethyl-pyrazine). No pyrazines were identified in the resultant raw milks in that same study (5). Pyrazine was not identified in any feed or rumen sample in this study (Supplementary Table 1). Maltol is a known Maillard end product from lactose and/or lysine residues (38, 39) and has previously been identified in heat-treated milks and has been described as a potent sweet aromatic compound (40). Maltol is derived from the metabolism of dietary sugars such as maltose, and was not identified in any feed or rumen sample in this study (Supplementary Table 1), and, as the milk did not undergo thermal treatment, it is difficult to discern the source of maltol, but it may have been created by microbial activity in the raw milk. 3-/4-Ethylphenol (tentative identification) is a phenolic compound that has been previously been identified in bovine milk (41) and is likely a result of isoflavone (formononetin, biochanin A, and genistein) or amino acid metabolism in the rumen (42, 43) but also potentially in the milk. A previous study by Faulkner et al. (6) found higher levels of 4-ethylphenol in TMR feed than in pasture, but it was not detected in the milk from these feeding systems, which is in agreement with the results of this study whereby 3/4-ethylphenol was detected in both GRS and TMR feed (higher in TMR) but not detected in the rumen or milk samples (Supplementary Table 1). 4-ethylphenol has a very characteristic horse stable-like, faecal, and medicinal aroma, while 3-ethylphenol has a leather-like and ink-like aroma (44).

Only one lactone γ-nonalactone was significantly different in these milks and at higher abundances in TMR milk. γ-Nonalactone has been described as having a coconut, peach-like aroma and is very odour active (45). Lactones such as γ-nonalactone are fat-derived aroma compounds and are generally formed through thermal degradation of γ-hydroxy acids (46), or *via* β-oxidation of hydroxy acids followed by cyclisation (27), or by one-step non-enzymatic reactions (47). Ueda et al. (33) proposed that diets, which included grains, meals, and oats, resulted in greater abundances of some lactones (γ-dodecalactone and δ-dodecalactone) in the resultant milks, as these diets induced propionate (a hydroxycarboxylic acid) metabolism in the rumen. Villeneuve et al. (27) found higher levels of some γ-lactones in milk from hay-fed cows than in cows-fed silage or pasture. They also suggested that unsaturated fatty acids may be transformed by hydration to intermediate hydroxyl acids in the rumen and subsequently create lactones through oxidation and cyclisation that end up in animal tissue and milk. γ-Nonalactone was present in all feed and rumen samples in this study (Supplementary Table 1), suggesting its potential direct transfer from the diet, and abundances were much higher in TMR feed than GRS feed (but abundances were similar in the rumen samples). Bendall et al. (13) also found an odour-active lactone (γ-dodec-cis-6,cis-9-dienolactone) present in milk derived from a TMR diet but absent in cows on a pasture diet. However, it appears that the extraction method can also have a significant impact on the recovery of many lactones (20, 21) and that HS-SPME is not very suitable for the extraction of lactones (dependent upon fibre type), an extraction technique widely used for VOC analysis in bovine milk (27, 28, 31, 40).

### Key Aroma Active Volatiles Identified by Odour Intensity Analysis

In total, 44 distinct odour activities were perceived by the panellists in raw bovine milks (Table 3); most were from individual VOC, but eight consisted of two or three co-eluted VOC, and six remain unidentified (UNC), which is not uncommon when VOC is present at a concentration above their odour threshold but below the limit of MS detection. The total OI values for GRS and TMR milk were 61.2 and 66.2, respectively. The higher OI of TMR milk likely reflects the greater number of odour-active VOC and is likely influenced by the more diverse composition of the TMR diet. Only 27 of the 44 odour activities were present in both sample types, highlighting the diversity of aromas between both samples derived from the GRS and TMR diets. In summary, 34 VOC were perceived in raw GRS milk compared to 36 VOC in raw TMR milk. For the purpose of this study, only odour activities with an average OI of ≥ 2. are discussed in detail (Table 3), which corresponds to 20 distinct odour activities between both raw GRS and raw TMR milk.

**Table 3:** Aroma-active compounds perceived in raw bovine milk from cows-fed grass (GRS) or TMR by gas-chromatography olfactometry (GC-O).

	**Retention index**						**AEDA FD values**		**Odour intensity (OI)**			
**Order of identification**	**(RI ^**a**^) DB-624 UI** **(volatile TD analysis)**	**(RI ^**b**^) DB-624 UI** **(GC-O analysis)**	**(LRI ^**c**^) LRI (literature)**	**Identification method**	**Compound**	**Aromas perceived by panellists**	**Raw GRS milk**	**Raw TMR milk**	**Raw GRS milk**	**Raw TMR milk**	**Odour threshold (ppb)**	**Odour threshold reference**
1	460	-	458	A, C	Methanethiol	Fishy, cabbage	50	10	1.4	1.6	1	Devos et al. (48)
2	593	582	629	A, C	2-Methylpropanal	Sweet, fresh	0	2	-	1.2	0.1–2.3	Leffingwell and Associates et al. (49)
3	631	574	630	A, C	2,3-Butanedione	Fresh, sweet, caramel, butterscotch, biscuity, baked	2	20	2.4	2.2	2.3–6.5	Leffingwell and Associates et al. (49)
4	686	685	699	A, C	Acetic acid	Vinegar	5	5	2.2	2.1	480–1,000	New Jersey Department of Health et al. (50)
5	779	755.4	785	A, C	Dimethyl disulfide	Musty, cardboard, sulphur, fishy	10	10	1.4	1.3	0.16-12	Leffingwell and Associates et al. (49)
6	781	764	784	A, C	Methyl Isobutyl Ketone	Sweet	1	0	0.9	-	-	-
7	794	774	796	A, C	Toluene	Musty, damp, earthy, plastic	20	10	1.9	1.4	4,680	Leonardos et al. (51)
	815	810	812	A, C	1-Pentanol	plastic					4,000	Leffingwell and Associates et al. (49)
8	-	830	837	A, C	Hexanal	Roasted, fresh, floral, herbal, vegetable	10	0	1.4	-	4.5-5.0	Leffingwell and Associates et al. (49)
9	866	841	868	B, C	2-Methylpropanoic acid	Fruity, citrus, fatty, roast chicken, cheesy	5	5	1.5	1.5	8100	Leffingwell and Associates et al. (49)
10	862	878	859	A, C	Butanoic acid	Cheesy, dairy, buttery	20	5	2.3	2.5	240	Leffingwell and Associates et al. (49)
11	899	892	901	A, C	Furfural	Cheesy, sour, sour milk, dairy, nutty, bready, baked, roasted	50	50	2.2	2.4	16,000	Franco et al. (52)
12	927; 936	929; 931	926.6; 931.5	B, C; A, C	2-Furanmethanol, 2-Heptanone	Barnyard, animal, musty, bready, cheesy	10	5	1.5	2.2	8,000; 14–3000	U.S. National Library of Medicine and National Centre for Biotechnology Information et al. (53)
13	914; 922; 945	932; 937; 938	917; 935; 939	A, C; A, C; A, C	3-Methylbutanoic acid; 2-Methylbutanoic acid; Heptanal	Buttery, animal, barnyard, nutty, bready	10	5	1.8	2.8	120–170; 3–360	Leffingwell and Associates et al. (49); Burdock et al. (54)
14	-; -	950; 959	952; 963	A, C; A, C	2,5-Dimethylpyrazine; 2,3-Dimethylpyrazine	Smokey, barnyard, animal, roasted, toasted, cooked potato	20	50	2.0	2.6	800–1,800; 2,500–35,000	Leffingwell and Associates et al. (49)
15	-	1009	1014	A, C	2-Pentylfuran	Roasted, toasted, bready, potato, popcorn	20	50	2.7	2.5	6	Leffingwell and Associates et al. (49)
16	-	-	-	C	Unidentified 1	Cooked potato, roasty, musty	1	1	2.3	1.9	-	-
17	-	1010	-	C	Unidentified 2	Fishy, salty, stale, sulphur, chemical, woody, cabbage	10	10	1.6	1.7	-	-
18	1034; 1031; 1028	1020; 1025; 1027	1032; 1027; 1024	A, C; A, C; A, C	Benzaldehyde; Butyrolactone; Ethyl hexanoate	Sweet, caramel, herbal, fruity, cherry	50	50	1.9	2.5	350–3,500; 1,000; 30	Leffingwell and Associates et al. (49); Poisson and Schieberle et al. (55); Guo et al. (56)
19	-	1028	1023.7	B, C	1-Octen-3-ol (tentative)	Green, fresh, earthy, mushroom	50	50	1.7	2.0	1	Leffingwell and Associates et al. (49)
20	-	1044	-	C	Unidentified 3	Green, floral, fresh, grassy, earthy	20	10	1.9	2.1	-	-
21	1040	-	1037.5	A, C	Isomaltol	Sweet, cotton candy, fruity, aniseed, medicinal	20	10	1.3	1.6	0.002	Cliff et al. (57)
22	1051	1096	1052	A, C	Hexanoic acid	Cheesy, smokey, bready, roasted	1	0	1.2	-	3,000	Leffingwell and Associates et al. (49)
23	1077	1079	1079	A, C	2-Ethylhexanol	Sweet, solvent	1	0	1.0	-	27,0000	Leffingwell and Associates et al. (49)
24	1120	1108	1121	B, C	Benzeneacetaldehyde	Pungent, cleaning agent, musty	0	1	-	1.2	4	Liu et al. (58)
25	1117	1120	1124	A, C	1-Octanol	Mushroom, stale, damp	0	1	-	1.2	110–130	Leffingwell and Associates et al. (49)
26	-	-	-	C	Unidentified 4	Animal, pungent, smokey, burnt, eggy	50	50	2.5	1.2	-	-
27	1151	1148	1151	A, C	Nonanal	Solvent, fresh, artificial, chemical	10	0	1.4	-	1	Leffingwell and Associates et al. (49)
28	-	1163	1166	A, C	γ-Hexalactone	BBQ, caramel, tobacco, toasted, toffee	20	2	2.3	1.8	1,600	Leffingwell and Associates et al. (49)
29	1182	1170	1171	B, C	Methyl 2-furoate	Toffee, fruity, sweet, caramel	0	20	2.3	2.2	NF	-
30	-	1078	-	C	Unidentified 5	Fresh, herbal, sweet	50	50	2.2	2.4	-	-
31	-	1142	-	C	Unidentified 6	Sweet, floral	50	0	2.5	-	-	-
32	1193	1193.6	1193	A, C	Phenylethyl alcohol	Sweet, herbal, fruity, spicy	50	20	2.3	2.7	65	Leffingwell and Associates et al. (49)
33	1193	1193	1204	A, C	Maltol	Caramel, sweet, cotton candy	100	10	1.6	2.3	2,600	Poisson and Schieberle et al. (55)
34	1193; 1196	1195	1183.5; 1192	A, C; A, C	p-Cresol; 2-Pyrrolidone	Barnyard, pungent, animal, solvent	20	50	1.6	2.6	1 NF	Leonardos et al. (51)
35	1222	1226	1224	A, C	Ethyl octanoate	Solvent, aldehydic, alcohol	20	0	1.8	-	147	Poisson and Schieberle et al. (55)
36	1254	1252	1256	A, C	Decanal	Solvent, mushroom, animal, floral, grassy	0	10	-	2.3	0.1	Leffingwell and Associates et al. (49)
37	1242	1270	1245	A, C	Octanoic acid	Smokey, toasted, animal, burnt milk	0	10	-	1.0	10,000	Peinado et al. (59)
38	1284	1286; 1286; 1285	1279; 1279; 1284	B, C; B, C; A, C	3-Ethylphenol (tentative); 4-Ethylphenol (tentative); Benzoic acid	Smokey, animal, burnt milk	0	50	-	1.6	1.7–800; 1–600	Czerny et al. (60); Dietz and Traud et al. (61)
39	1322	1298	1315	A, C	Benzothiazole	Smokey, roasted, caramel	0	10	-	1.6	80	Leffingwell and Associates et al. (49)
40	1320	1312	-	B, C	2-Phenoxyethanol	Sweet, burnt	0	1	-	1.6	NF	-
41	1345; 1411	1343	1352; 1408	A, C; A, C	2-Undecanone; 2-Methoxy-4-vinylphenol	Aniseed, sweet, herbal	2	5	0.8	1.3	7; 3	Leffingwell and Associates et al. (49)
42	1451	1460	1452	A, C	Hydrocinnamic acid	Sweet, floral, creamy	10	0	0.7	-	NF	-
43	-	1485	1489	A, C	γ-Nonalactone	Sweet, caramel, burnt, lactone	0	1	-	0.4	65	Siek et al. (62)
44	1547; 1635; 1724	1546; 1644; 1724	1547; 1641; 1725	B, C; A, C; A, C	2-Tridecanone; Dodecanoic acid; Tetradecanol	Smokey, herbal	2	5	0.7	0.7	NF; 10,000; NF	Leffingwell and Associates et al. (49)
	Total odour intensity	61.2	66.2									

a *Retention index (RI) calculated from GC-O results on a DB-624 UI column*.

b *Retention index (RI) calculated from thermal desorption (TD) results on a DB-624 UI column*.

c *Retention index found in the literature (LRI) for a DB-624 UI column*.

*Identification method (IM): A: identification based on comparison to the NIST mass spectral database, RI values from the literature, and an in-house library created using authentic compounds with target and qualifier ions and linear RI for each compound; B: tentative identification using RI values and LRI matching; C: identification with GC-O; NF: not found; FD: factor dilution*.

However, it is worth noting that seven odour activities with OI < 2. were detected in raw GRS milk, but not in raw TMR milk; ethyl octanoate (OI, 1.8), nonanal (OI, 1.4), hexanal (OI, 1.4), hexanoic acid (OI, 1.2), 2-ethylhexanol (OI, 1.), methyl isobutyl ketone (OI, 0.9), and hydrocinnamic acid (OI, 0.7).

Similarly, further eight odour activities with OI < 2. were detected in raw TMR milk but not in raw GRS milk: 3/4-ethylphenol (tentative identification)/benzoic acid (OI, 1.6), benzothiazole (OI, 1.6), 2-phenyoxyethanol (OI, 1.6), 2-methylpropanal (OI, 1.2), benzeneacetaldehyde (OI, 1.2), 1-octanol (OI, 1.2), octanoic acid (OI, 1.), and γ-nonalactone (OI, 0.4).

#### Aroma-Active Volatiles in Raw GRS Milk by Odour Intensity Analysis

**Figure 1:**
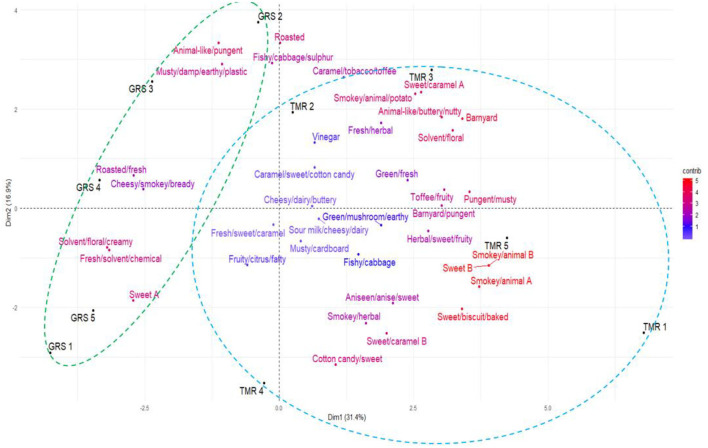
Principal component Biplot analysis of the odour descriptors perceived by the five panellists based on odour intensity values for raw GRS and total mixed ration (TMR) milk. Colour gradient: low = white, mid = blue, high = red, midpoint set at 1.

**Figure 2:**
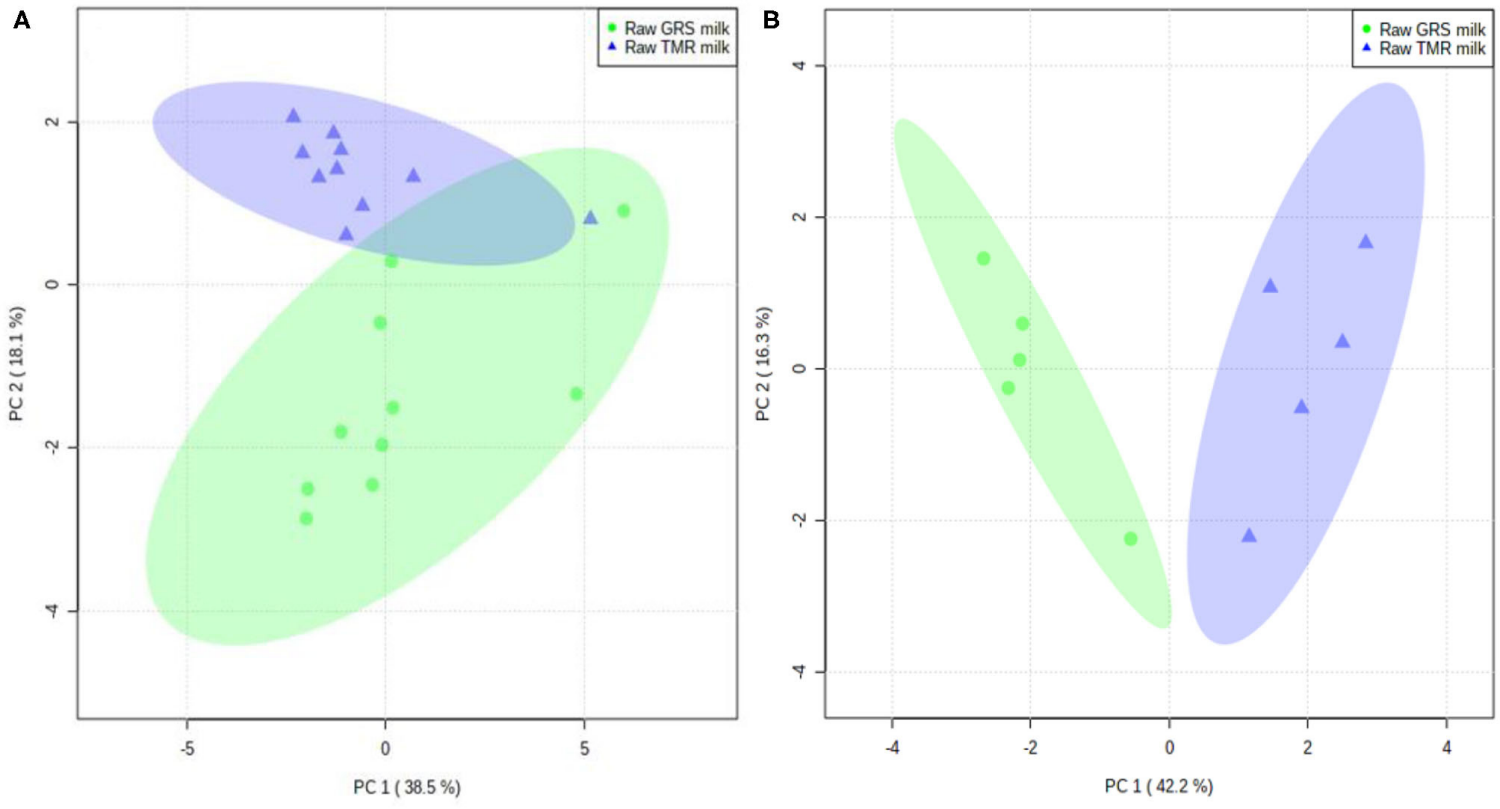
Principal component analysis (PCA) of the odour intensities perceived by panellists based on odour intensity values for raw GRS and TMR milk. **(A)** replicate data (*n* = 10); **(B)** averaged data for each panellist (*n* = 5).

Thirteen distinct odour activities dominated the aroma of raw GRS milk (OI ≥ 2.); four of which were unidentified (UNC 1, UNC 4, UNC 5, and UNC 6). Based on their OI, the order of their potential significance was as follows: 2-pentylfuran (roasted, toasted, bready, potato, popcorn; OI, 2.7), UNC 4 (animal, pungent, smokey, burnt, eggy; OI, 2.5), UNC 6 (sweet, floral; OI, 2.4), 2,3-butanedione (fresh, sweet, butterscotch, biscuity, baked; OI, 2.4), butanoic acid (cheesy, dairy, buttery; OI, 2.3), UNC 1 (cooked potato, roasty, musty; OI, 2.3), γ-hexalactone (barbeque, caramel, tobacco, toasted, toffee; OI, 2.3), methyl 2-furoate (toffee, fruity, sweet, caramel; OI, 2.3), phenylethyl alcohol (sweet, herbal, fruity, spicy; OI, 2.3), acetic acid (vinegar; OI, 2.2), furfural (cheesy, sour, sour milk, dairy, nutty, bready, baked, roasted; OI, 2.2), UNC 5 (fresh, herbal, sweet; OI, 2.2), and 2,5-dimethylpyrazine/2,3-dimethylpyrazine (smokey, barnyard, animal, roasted, toasted, cooked potato; OI, 2.). Figures 1, 2 illustrate the odour descriptors perceived by the 5 panellists for raw GRS and TMR milk.

2-Pentylfuran is likely a Maillard reaction product, which has been associated with caramel odours (30). As it was present in all samples in this study, it is likely derived directly from the diet (Supplementary Table 1). Clarke et al. (5) also found 2-pentylfuran in feed but not in the resultant milk. These authors also found it at higher abundances in GRS and GRS/CLV than in the TMR feed. However, in another study, 2-pentylfuran was found at the greatest abundance in TMR feed; although still present in GRS, GRS/CLV feed, it was only present in raw milk derived from a TMR diet and was not present post pasteurisation (4). 2-Pentylfuran was present in both raw milk samples in this study. 2,3-Butanedione (diacetyl) is quite an odour active and described as having a buttery, pastry aroma (10), not that dissimilar to this study (fresh, sweet, butterscotch, biscuity, baked). 2,3-butanedione is a common VOC in dairy products produced predominately by pyruvate metabolism. Faulkner et al. (6) found 2,3-butanedione in TMR feed but not in GRS or GRS/CLV feed, and was absent in pasteurised bovine milk derived from these diets. Previous studies have found 2,3-butanedione at a greater abundance in raw milk derived from hay than from maize or grass silage (35), and in raw milk from TMR and pasture (11). In this study, 2,3-butanedione was present in all feed and rumen samples (Supplementary Table 1) and, therefore, is also likely directly transferred from the diet. As mentioned, short chain fatty acids such as butanoic acid are primarily produced by *de novo* synthesis in the mammary gland, which is impacted by diet but can also be directly transferred from the diet in free form (1), or from lipolysis (36). Butanoic acid was described as cheesy, dairy, buttery, and was present in all feed and rumen samples in this study (Supplementary Table 1). It was at very high but similar abundances in both GRS and TMR rumen samples. Other studies have also identified butanoic acid in bovine milk (1, 13, 16, 27, 28), but only Bendall et al. (13) (vomit: feta cheese) and Ai et al. (15) (green) found it to be odour active. Clarke et al. (5) and Faulkner et al. (6) found butanoic acid in GRS, GRS/CLV, and TMR feed, with significantly more in TMR feed. Clarke et al. (5) did not find butanoic acid in fresh raw milk produced from these diets, but Faulkner et al. (6) did, although abundances were not statistically different in raw or pasteurised milk from these diets. The choice of the extraction method and the GC column used is likely to have a significant effect on the recovery of butanoic acid, as the recovery of acids is greatly influenced by the polarity of the sorbent and GC column (21). γ-Hexalactone is a lactone, with a coconut, fruity, sweet aroma and a medium odour threshold (45), but was described in this study as having a barbeque, caramel, tobacco, toasted, toffee aroma (as the odour was so diverse, it is possible that co-elution may have occurred with another odour-active VOC that was below the limits of MS detection). As previously mentioned, lactones are potentially produced from several different routes. Clarke et al. (5) found γ-hexalactone in GRS, GRS/CLV, and TMR feed (statistically higher abundances in GRS/CLV) but not in raw milk derived from these diets. In this study, γ-hexalactone was found in both GRS and TMR feed, but not in any rumen samples.

Methyl 2-furoate is a furan product of the Maillard reaction (63) and has also been found in bovine milk derived from crop silage/hay (64). It has a sweet, caramel brown sugar musty aroma (65) and was described similarly (toffee, fruity, sweet, caramel) in this study. It was not present in any feed or rumen samples in this study (Supplementary Table 1) and, therefore, unlikely to derive directly from the diet. Phenylethyl alcohol (2-phenylethanol) has been previously found in GRS, GRS/CLV, and TMR feed samples but not in any raw or pasteurised milks derived from these feeds (5, 6). It was described as having a slightly rose-like aroma and has previously been found in raw milk (12). Its aroma description was quite different in this study (sweet, herbal, fruity, spicy), and may also indicate co-elution with an odour-active VOC that is below the limits of MS detection. It is likely a product of Strecker degradation of phenylalanine (66), and was present in all of the feed and rumen samples in this study (Supplementary Table 1), highlighting potential direct transfer from the diet. Acetic acid has previously been found in milk (6, 13, 15, 27, 28). Similar to other short chain acids, acetic acid is thought to directly transfer from the diet into milk (4), and this seems to confirm results in this study where acetic acid was found in all feed and rumen samples, with quite high abundance in the rumen samples (Supplementary Table 1). Faulkner et al. (6) found highest abundance of acetic acid in TMR feed, but subsequently highest levels in GRS raw milk but not statically different in pasteurised milk from GRS, GRS/CLV or TMR. Acetic acid has a vinegar aroma and matches that found in this study but is actually not that odour active (13). Again it is worth mentioning like all acids recovery of acetic acid is particularly impacted by the extraction technique. Furfural is commercially produced from lignocellulose biomass (67), so is likely to either be directly present in forage or from metabolised lignin. In this study, furfural was present in both GRS and TMR feed (at higher levels in TMR feed) and in both GRS and TMR RF (Supplementary Table 1). Furfural has a distinct barny/brothy aroma (40) and was described as cheesy, sour, sour milk, dairy, nutty, bready, baked, roasted in this study (as mentioned earlier, the diverse odour descriptors likely indicate co-elution with another odour-active VOC that is below the limits of MS detection). Clarke et al. (5) found furfural at a higher abundance in GRS than in GRS/CLV or TMR feed but not in raw milk derived from these diets. 2,5-dimethylpyrazine/2,3-dimethylpyrazine were more odour active in raw GRS milk. Both pyrazines were present in TMR feed only, and 2,3-dimethylpyrazine was not present in any rumen sample but was present in both raw milks (GRS and TMR) (Supplementary Table 1). 2,5-Dimethylpyrazine was present in GRS and TMR RF but at higher levels in TMR RF; however, it was absent in raw GRS milk. Clarke et al. (5) found 2,3-dimethylpyrazine in GRS, GRS/CLV, and TMR feed, with statistically higher levels in TMR feed. These authors did not find any pyrazines in raw or pasteurised milk from these feeds. As stated, pyrazines can be formed *via* the Strecker reaction enhanced by heat treatment (35) *via* Maillard browning or microbial metabolism (37). Mounchili et al. (14) found 2,3-dimethylpyrazine with 2,6-dimethylpyrazine in milk and described them as having intense, roasted breadcrumbs, bake house, cooked rice, biscuits, cooked milk aroma. In this study, 2,5-dimethylpyrazine and 2,3-dimethylpyrazine were described as having a smokey, barnyard, animal, roasted, toasted, cooked potato aroma.

#### Odour Active Volatile in Raw TMR Milk by Odour Intensity Analysis

The odour of raw TMR milk was dominated by 17 distinct sets of aromas with OI ≥ 2.; some of which consisted of co-eluted VOC and two UNC compounds (UNC 3 and UNC 5). The key odours were as follows in order of perceived intensity: 3-methyl butanoic acid/2-methyl butanoic acid/heptanal (buttery, animal, barnyard, nutty, bready; OI, 2.8), phenylethyl alcohol (sweet, herbal, fruity, spicy; OI, 2.7), p-cresol/2-pyrrolidone (barnyard, pungent, animal, solvent; OI, 2.6), 2,5-dimethylpyrazine/2,3-dimethylpyrazine (smokey, barnyard, animal, roasted, toasted, cooked potato; OI, 2.6), 2-pentylfuran (roasted, toasted, bready, potato, popcorn; OI, 2.5), benzaldehyde/γ-butyrolactone/ethyl hexanoate (sweet, caramel, herbal, fruity, cherry; OI, 2.5), butanoic acid (cheesy, dairy, buttery; OI, 2.5), furfural (cheesy, sour, sour milk, dairy, nutty, bready, baked, roasted; OI, 2.4), decanal (solvent, mushroom, animal, floral, grassy; OI, 2.3), maltol (caramel, sweet, cotton candy; OI, 2.3), 2,3-butanedione (fresh, sweet, butterscotch, biscuity, baked; OI, 2.2), 2-furanmethanol/2-heptanone (barnyard, animal, musty, bready, cheesy; OI, 2.2), methyl 2-furoate (toffee, fruity, sweet, caramel; OI, 2.2), acetic acid (vinegar; OI, 2.1), UNC 3 (green, floral, fresh, grassy, earthy; OI, 2.1), and 1-octen-3-ol (tentative identification; green, fresh, earthy, mushroom; OI, 2.). Figures 1, 2 illustrate the odour descriptors perceived by the 5 panellists for raw GRS and TMR milk.

The most odour-active aroma was generated from 3-methyl butanoic acid/2-methyl butanoic acid /heptanal and described as having a buttery, animal, barnyard, nutty, bready aroma (with co-elution, it is difficult to discern which VOC are having the greatest aroma impact). As mentioned previously, branched chain acids, such as 3-methyl butanoic acid and 2-methyl butanoic acid, are products of Strecker degradation. 2-Methylbutanoic acid was present at significantly higher levels in raw TMR milk but was not present in any feed or rumen samples in this study (Supplementary Table 1). However, Faulkner et al. (6) identified it in TMR feed but not in GRS or GRS/CLV feed, and these same authors did not find it in raw or pasteurised milk from these feeds. It has previously been described as having a fruity, sweaty, rancid, burnt, sour aroma in milk (14). 3-Methyl butanoic acid was present in every sample in this study, except in raw milk from GRS or in the GRS feed (Supplementary Table 1). Thus, it was not present in the feed but was present in GRS RF. It is likely metabolised into corresponding alcohols and esters, which may account for its absence in the raw GRS milk. It has also been found in raw milk and has been described as having a slightly grassy, cooked vegetable aroma (13). Heptanal is a major lipid oxidation product in milk (5) and has been widely identified in milk described as cheesy caramel (13) or green sweet (10). Heptanal was present in all samples in this study (Supplementary Table 1) and thus appears to be both directly transferred from diet and derived from lipid oxidation. The next most active aroma – sweet, herbal, fruity, spicy – was due to phenylethyl alcohol, which has already been identified as a very abundant VOC in raw GRS milk in this study, and likely also derived directly from the diet. It is noteworthy that it is impacting the aroma of both milks. The next most significant aroma – barnyard, pungent, animal, solvent – was generated by co-eluting peaks of p-cresol/2-pyrrolidone. p-Cresol is a degradation product of β-carotene and isoflavones (5, 6). In this study, p-cresol was identified in all feed, rumen, and milk samples but was one of the most abundant VOC found in rumen samples (Supplementary Table 1). A higher abundance of p-cresol was found in TMR feed, but slightly higher levels were found in raw GRS milk than raw TMR milk. p-Cresol is thought to be one of the main VOC behind the barnyard aroma often perceived in pasture-produced dairy products (6), and, therefore, it is interesting to note that it appears to have a greater contribution to the aroma of raw TMR milk in this study, although was co-eluting with 2-pyrrolidone. The odour activity of p-cresol is at an intermediate level in comparison to most VOCs (13). 2-Pyrrolidone is a lactam cyclisation product of γ-amino butyric acid (68) and, thus, may be formed in the rumen but also appears to be present in both GRS and TMR feed (Supplementary Table 1). However, it was only present in the GRS rumen samples and was in greater abundance in raw GRS milk. Despite the fact that its abundance is lower than in raw GRS milk, it appears more easily perceived in raw TMR milk (although it did co-elute with p-cresol). 2-Pyrrolidone is a VOC that has not been previously identified in bovine milk.

Both 2,5-dimethylpyrazine/2,3-dimethylpyrazine were described as having the next most intense aroma – smokey, barnyard, animal, roasted, toasted, cooked potato – in this study. As mentioned already, these VOC were also identified as important odour-active compounds in raw GRS milk, which appear to both come directly from the diet, but also formed by microbial metabolism, Strecker reactions, or Maillard browning. It is possible that higher abundances in TMR milk were potentially due to direct transfer rather than other means based on the fact that neither was found in the GRS feed in this study (thus, not derived directly from GRS feed). The next most significant aroma was attributed to 2-pentylfuran, which was described as roasted, toasted, bready, potato, popcorn, somewhat different to caramel (30) and, again, may indicate co-elution with another odour-active VOC below the limit of MS detection. As mentioned, it is a product of the Maillard reaction and was also identified as an important VOC in raw GRS milk in this study but appears to be mainly derived directly from the diet.

A sweet, caramel, herbal, fruity, cherry aroma was derived from co-eluting VOC (benzaldehyde/γ-butyrolactone/ethyl hexanoate). Benzaldehyde has previously been described as having an almond-like nutty aroma and was found at low abundances in raw UHT bovine milk (12). Moio et al. (11) also found benzaldehyde in raw and pasteurised bovine milk, with higher abundances in UHT milk. However, these authors did not find that it contributed to the aroma of milk. Benzaldehyde has also previously been found in raw milk derived from pasture, hay, or silage (27). Faulkner et al. (6) also found significantly similar abundances in GRS, GRS/CLV, and TMR feed (6) and raw milk derived from GRS but not from GRS/CLV or TMR or in any milk post pasteurisation. Benzaldehyde is thought to derive from the metabolism of phenylalanine (4), which likely occurs in the rumen but also appears to be directly transferred from the diet as all feed, rumen, and milk samples in this study contained benzaldehyde (Supplementary Table 1). γ-butyrolactone is another lactone and has a creamy, oily, subtle fatty aroma (4). It was not present in any fed or rumen samples in this study (Supplementary Table 1), which is similar to that found by Faulkner et al. (6). However, these authors did find it in pasteurised milk produced from TMR, but not in pasteurised milk from GRS or GRS/CLV, or in raw milk produced from any of these feeds. Ethyl hexanoate has a fruity, malty, young cheese, mouldy aroma and is derived from the esterification of ethanol and hexanoic acid but is also directly transferred from feed (4). Ethyl hexanoate was present at a high abundance in TMR feed but absent in GRS feed in this study (Supplementary Table 1). Ethyl hexanoate has previously been found to be one of the most abundant esters in raw bovine milk but absent post pasteurisation (11, 12); however, more importantly, it was thought not to influence the aroma of raw bovine milk (11). Ethyl hexanoate was also found to be quite abundant in raw milk from cows-fed hay, silage, or pasture (27), and Clarke et al. (5) found ethyl hexanoate in GRS, GRS/CLV, and TMR feed but absent in the subsequent raw and pasteurised milks derived from these diets.

Butanoic acid also contributed to the aroma of raw TMR milk, but, as previously mentioned, also contributed to the aroma of raw GRS milk. Butanoic acid appears to be derived directly from the diet through *de novo* synthesis and lipolysis (Supplementary Table 1) (1, 36). As mentioned, furfural, 2,3-butanedione, and acetic acid also contributed to the aroma of raw GRS milk and are also likely derived from the diet. Methyl-2-furoate also contributed to the aroma of raw GRS milk but did not derive from the diet. Decanal is likely a result of lipid oxidation and directly transferred from the diet and has previously been identified in raw bovine milk (5, 6, 11, 14). It was described as having a solvent, mushroom, animal, floral, grassy aroma in this study and was present in all feed, rumen, and raw milk samples (Supplementary Table 1) but only influenced the aroma of raw TMR milk. The broad description likely also indicates co-elution with another aroma-active VOC that is present below the limits of detection of the MS. Maltol did influence the aroma of raw GRS milk (OI, 1.6) but was not discussed as levels were below OI, 2. As mentioned, it did not directly derive from diet but may be a product of microbial activity in raw milk. 2-furanmethanol was only identified in milk samples and is a result of Maillard reactions between an amino acid and sugar, or from oxidation of poly-unsaturated fatty acids (7). The higher abundance in raw TMR milk and corresponding higher aroma intensity is consistent with the findings by Faulkner et al. (6). 2-Heptanone is a secondary oxidation product commonly found in dairy products (6, 69), and was identified in all feed, RF, RB, and raw milk samples in this study, thus likely derived from the diet. 1-Octen-3-ol (tentative identification based on published odour references and LRI) is a product of lipid oxidation and likely influenced by the abundances of specific unsaturated fatty acids in the milk due to the different bovine diets (4). In this study, it was described as having a green, fresh, earthy, mushroom aroma and did contribute to the aroma of both raw GRS and raw TMR milk (greater influence in raw TMR milk by OI). However, 1-octen-3-ol was not identified in either raw GRS or TMR milk by GCMS in this study, possibly due to co-elution and/or that the compound was present below its limit of detection by MS (it was only tentatively identified by olfactometry analysis due to low abundance).

Both UNC 3 and UNC 5 influenced the aroma of both raw TMR and GRS milk, with UNC 5 having a slightly greater OI value, and higher in raw TMR than raw GRS milk.

### Key Aroma-Active Volatiles Identified by AEDA

Aroma extraction dilution analysis can potentially provide more information on the significance of specific aromas than OI alone, as the extract can be diluted extensively in a very controlled manner using the split value in the GC injection port, which also negates any sample matrix effects. Therefore, aromas that can be perceived at the greatest dilution are likely to be the most significant in terms of aroma and flavour perception.

### Aroma Active Volatiles in Raw GRS Milk by AEDA

**Figure 3:**
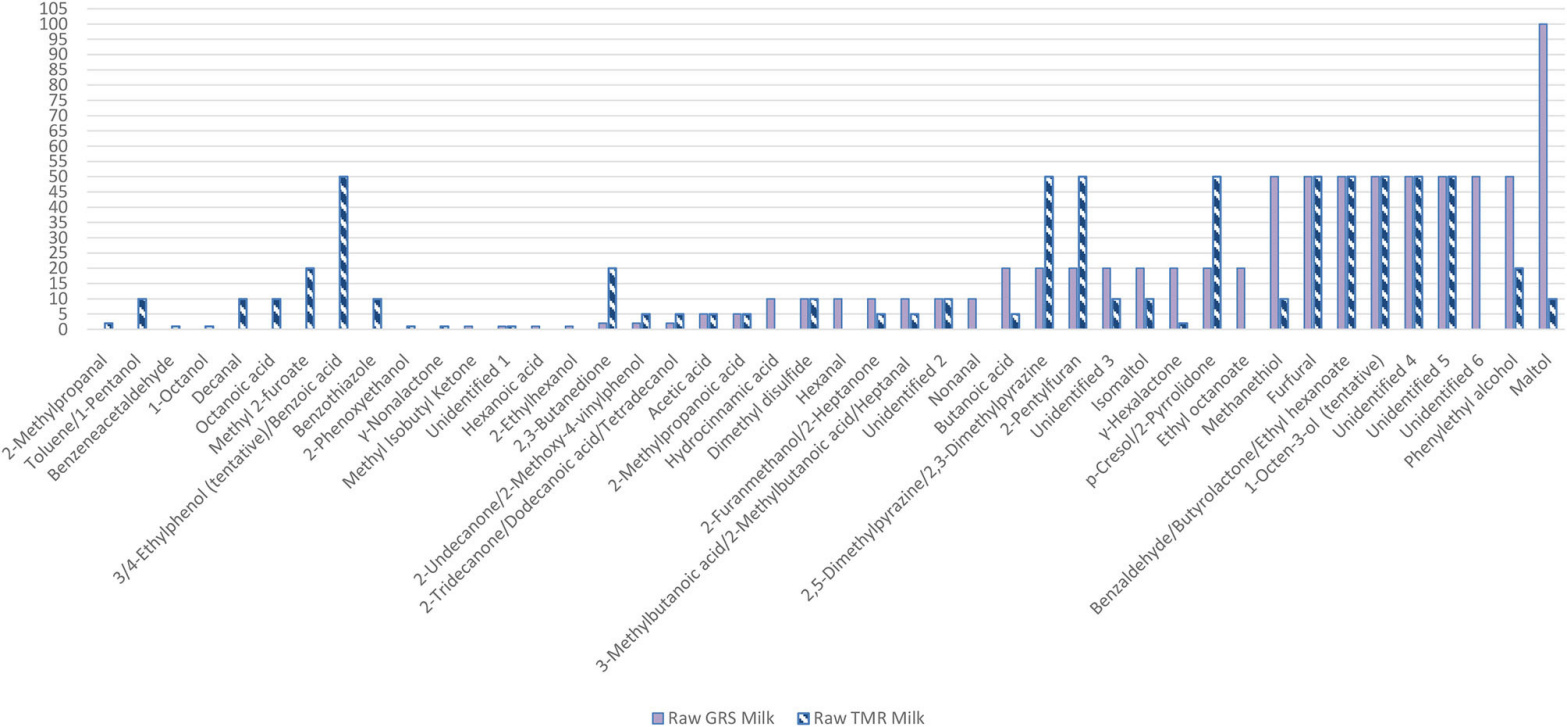
A bar chart illustrating the aroma extraction dilution analysis (AEDA) factor dilution values for the 44 volatile organic compounds identified *via* gas-chromatography olfactometry in raw grass milk (GRS) and raw TMR milk; range: 0–100. The higher the FD, the more odour intense the compound is.

From the AEDA study based on the FD values (Table 3, Figure 3), the primary VOC contributing to the overall aroma of raw GRS milk were maltol (FD100), with each of the following having identical FD values; methanethiol (FD50), furfural (FD50), benzaldehyde/γ-butyrolactone/ethyl hexanoate (FD50), 1-octen-3-ol (FD50), phenylethyl alcohol (FD50), and 3 unidentified VOC; UNC 4(FD50), UNC 5 (FD50), and UNC 6 (FD 50).

As previously stated, Maltol has a sweet odour and is a product of the Maillard reaction (39), and was described as having a caramel, sweet, cotton candy aroma in this study and has been previously been identified in bovine milk (28, 38, 39). As stated, it appears to arise from dietary sugars as it was not present in any of the feed or rumen samples. Oddly, even though it was present at significantly greater abundances in raw TMR milk than in raw GRS milk, it appears to have a greater impact on the aroma of the raw GRS milk, although, from the OI study, it was deemed potentially more important to the aroma of raw TMR milk. Methanethiol was described as having a fishy, cabbage aroma; although it has previously been described as having an intense potato soup or cooked potato aroma (67), this discrepancy may also be due to co-elution with another odour-active VOC that is below the limits of MS detection. It was present in every sample in this study (Supplementary Table 1). It is thought to arise from methionine and is influenced by the application of heat (34); however, it is also readily oxidised to dimethyl disufide and dimethyl trisulfide (34). Clarke et al. (5) also found methanethiol in GRS, GRS/CLV, and TMR feed but not in raw milk derived from these diets. It was also identified as odour active by OI, but, as values were below OI 2., it was deemed of less significance; however, it is well recognised as a very odour active VOC (34). Furfural, as mentioned, was already identified as a key odour-active compound in raw GRS milk and raw TMR milk by OI in this study (slightly higher in raw TMR milk by OI), and is likely derived directly from the diet, although may be co-eluting with another odour-active VOC not detected by the MS.

A sweet, caramel, herbal, fruity cherry aroma was described for 3 co-eluting VOC, benzaldehyde, γ-butyrolactone, and ethyl hexanoate. As stated, these VOCs have different potential sources from amino acid metabolism (benzaldehyde), diet (benzaldehyde, ethyl hexanoate), lipid oxidation (γ-butyrolactone), and a combination of lipolysis and esterification (ethyl hexanoate) in milk. This same aroma from these co-eluting VOC was also found to be aroma active in both raw milks by OI but with a greater contribution than in raw GRS milk.

1-Octen-3-ol (tentative identification based on published odour references and LRI) as previously stated is a product of lipid oxidation, with a similar FD value in raw GRS and TMR milk. It was also identified as aroma active by OI (with a slightly higher importance in raw TMR milk). Phenylethyl alcohol had a sweet, herbal, fruity, spicy aroma (but, as previously stated, may be co-eluting with an odour-active VOC not detected by the MS) and was also identified as aroma active by OI, with a slightly greater influence for raw TMR milk. It appears to derive mainly from the diet, although it is also a product of Strecker degradation and was also like furfural previously identified as an important aroma VOC in raw GRS milk by OI. Three UNC VOC were also found to influence the aroma of raw GRS milk: UNC 4 (animal, pungent, smokey, burnt, eggy), UNC 5 (fresh, herbal, sweet), and UNC 6 (sweet, floral).

### Aroma Active Volatiles in Raw TMR Milk by AEDA

Nine aromas were found to influence the perception of raw TMR milk by AEDA; four of which were from co-eluted VOC and one from an unidentified VOC (Table 3, Figure 3). All of these VOC were perceived up to FD 50; furfural, 2,5-dimethylpyrazine/2.3-dimethylpyrazine, 2-pentyl furan, benzaldehyde/γ-butyrolactone/ethyl hexanoate, 1-octen-3-ol (tentative identification based on published odour references and LRI), UNC 4, UNC 5, p-cresol/2-pyrrolidone, and 3-/4-ethylphenol (tentative identification)/benzoic acid. The potential source and aroma of each of these VOC have already been discussed. As mentioned, 3-/4-ethylphenol is a phenolic compound that has been previously identified in bovine milk (2, 6, 41) and appears to be present in both raw GRS and TMR milk in this study. 3-/4-ethylphenol was identified in both feed samples being higher in TMR feed, which concurred with the results found by Faulkner et al. (6). This compound was not identified in the rumen or milk samples in this study (Supplementary Table 1), although likely present below its limit of detection. Benzoic acid is known to naturally occur in cultured dairy products from hippuric acid, phenylalanine, or the oxidation of benzaldehyde (70). It was not identified in any feed or rumen sample in this study (Supplementary Table 1). As it is not a common VOC in bovine milk and has a low-odour activity, it would appear unlikely to be contributing much to the aroma perceived in this study; therefore, 3-/4-ethylphenol was more likely to be impacting on odour activity of this aroma.

### Aromas Influenced by Diet as Detected by OI and AEDA

Eight VOC were perceived by panellists in raw GRS milk by OI and AEDA and not in raw TMR milk [UNC 6 (sweet, floral), ethyl octanoate (solvent, aldehydic, alcohol), hydrocinnamic acid (sweet, floral, creamy), nonanal (solvent, fresh, artificial, chemical), hexanal (roasted, fresh, floral, herbal, vegetable), hexanoic acid (cheesy, smokey, bready, roasted), 2-ethylhexanol (sweet, solvent), and methyl isobutyl ketone (sweet)]. Ethyl octanoate has previously been found in GRS, GRS/CLV, and TMR feed, with higher abundances in TMR (5, 6), but not in fresh raw milk derived from these feeds, although it did appear in raw milk derived from TMR after refrigerated storage (5). Ethyl octanoate was present in every feed, rumen, and milk sample in this study (Supplementary Table 1). Moio et al. (11) also found ethyl octanoate in raw milk derived from hay, and it has been described as having a floral aroma (12), somewhat different from the aroma described in this study (solvent, aldehydic, alcohol), which, as mentioned, may indicate that it is co-eluting with another aroma-active VOC that is below the limits of MS detection. As mentioned previously, ethyl esters are derived from short chain fatty acids primarily produced by *de novo* synthesis in the mammary gland but may also be derived directly from the diet (1).

Hydrocinnamic acid was identified only in raw milk samples in this study (Supplementary Table 1). It is a metabolite of phenylalanine degradation (71), and, thus, its higher abundance in GRS milk may be due to the higher protein content in pasture forage (31, 72). In this study, it was described as having a sweet, floral, creamy aroma. Nonanal has been found in GRS, GRS/CLV, and TMR feed (5, 6) and is commonly found in bovine milk (6, 11, 12, 27, 28, 73) as a result of an enzymatic breakdown or lipid oxidation. Nonanal was found in every sample in this study (Supplementary Table 1). Clarke et al. (5) previously found nonanal in GRS, GRS/CLV, and TMR feed, and at higher levels in the pasture diets and raw and pasteurised milk from each diet. It has been described as green, grass like fatty or tallow with a fatty odour (11, 12, 50), and as a solvent, fresh, artificial, chemical in this study. Again, the differences in odour descriptors may indicate co-elution with another aroma-active VOC present below the limits of MS detection.

Hexanal is commonly found in milk as a result of lipid oxidation of oleic, linoleic, and arachidonic acid (6, 74, 75), and differences in these fatty acid contents within the milks are, thus, likely influencing its abundance. Hexanal was present in both feed samples in this study but was not identified in any rumen samples (Supplementary Table 1). Even though abundances were considerably higher in GRS feed than TMR feed, abundances were similar in both raw milks in this study. Hexanal has also previously been identified in both GRS and TMR feed (5). Therefore, its presence in milk appears to be due to both lipid oxidation and direct transfer from the diet. It was identified in this study as having a cheesy, smokey, bready, roasted aroma, not that dissimilar to that described by Bendall et al. (13) as cooked. Hexanoic acid was present in every feed, rumen, and milk sample in this study (Supplementary Table 1) and always at higher levels in the TMR samples. Faulkner et al. (6) and Clarke et al. (5) also found high abundances of hexanoic acid in GRS, GRS/CLV, and TMR feed, but only Faulkner et al. (6) identified it in raw and pasteurised milks from these diets. Croissant et al. (28) found it in raw milk from both pasture and TMR diets, and Villeneuve et al. (27) found it in raw milk from hay, pasture, and silage diets, as did Coppa et al. (31) in milk from hay, rotational grazing, and continuous grazing. Previous studies have also found higher levels of hexanoic acid in raw milk produced from TMR (1). In this study, it was described as having a cheesy, smokey, bready, roasted aroma. Therefore, hexanoic acid is typically present at high abundances in bovine milk, and likely transfers directly from the feed, but also indirectly generated by *de novo* synthesis in the mammary gland (4). Again, as an acid, it is likely hugely influenced by the extraction method used.

Moio et al. (11) found 2-ethylhexanol in raw, pasteurised, and UHT milk but did not find that it impacted odour. However, Mounchili et al. (14) did find that it contributed to the odour of raw milk, although co-eluted with benzene acetaldehyde and was described as honey, vegetable, green, and moist. In this study, 2-ethylhexanol was described as having a sweet, solvent aroma. 2-ethylhexanol was present in every feed, rumen, and milk sample (Supplementary Table 1). It appears 2-ethylhexanol is likely derived directly from the diet as evident in this study. Methyl isobutyl ketone is likely a product of lipid oxidation (6) and imparted a sweet aroma in GRS milk in this study. Methyl isobutyl ketone was only present in the GRS feed, although was present in both GRS and TMR RB samples. While not significantly different, abundances were higher in GRS milk, similar to that found previously (6). Thus, it appears to derive from diet and lipid oxidation, although not a VOC, which has been commonly identified in raw milk to date.

Nine aromas associated with VOC or co-eluting VOC contributed to the aroma of raw TMR milk that did not contribute to the aroma of raw GRS milk [3-/4-ethylphenol (tentative identification)/benzoic acid (smokey, animal, burnt milk), decanal (solvent, mushroom, animal, floral, grassy), benzothiazole (smokey, roasted, caramel), octanoic acid (smokey, toasted, animal, burnt milk), 2-methylpropanal (sweet, fresh), 2-phenoxyethanol (sweet, burnt), benzeneacetaldehyde (pungent, cleaning agent, musty), 1-octanol (mushroom, stale, damp), and γ-nonalactone (sweet, caramel, burnt, lactone)]. The source and aroma of 3-/4-ethylphenol/benzoic acid, benzothiazole, and 2-methylpropanal have already been discussed in detail. Octanoic acid was described as having a smokey, toasted, animal, burnt milk aroma in this study, not that different from that described previously: burnt milk or pudding, intense (14). As mentioned, it was present in all samples in this study (Supplementary Table 1) and was consistently higher in TMR samples, thus, the likely reason it was perceived in raw TMR milk. 2-Phenoxyethanol, a phenolic compound, was identified as having a sweet burnt aroma in this study and was present in every feed, rumen, and milk sample in this study (Supplementary Table 1). Levels were not statistically different in raw GRS or TMR milk, and therefore, it is difficult to understand why it was only perceived in raw TMR milk. It is not a common VOC in bovine milk, although concentrations were previously found to be higher in evening milk compared to morning milk, and concentrations were shown to decrease over time, possibly due to oxidation (76).

Benzeneacetaldehyde (phenylacetaldehyde) is also a Strecker aldehyde (77) produced *via* phenalanine metabolism and has previously been reported in milk (14, 31). It was not identified in either feed in this study but was present in most rumen samples and both raw milks (Supplementary Table 1). It was described as having a pungent, cleaning agent, and musty aroma in this study, but is also likely further metabolised to acids, alcohols, and esters. Again, it is difficult to discern why it was only perceived in the raw TMR milk. 1-Octanol was present in every feed, rumen, and raw milk sample in this study (Supplementary Table 1). It was described as having a mushroom, stale damp aroma. Previous studies did not identify it in feed, but did in raw milk derived from CLV (6). However, other studies found it in both GRS and TMR feed, and raw milk from TMR (5). It is a product of lipid oxidation but likely also derived from feed. Again, it is difficult to discern why it would be perceived in raw TMR milk and not in raw GRS milk. γ-Nonalactone was found to be significantly (*p* =0.05) higher in TMR milk and was characterised as sweet, caramel, burnt, and lactone, but was not perceived in GRS milk. γ-Nonalactone was higher in TMR feed and TMR RB (Supplementary Table 1). As mentioned, lactones are naturally occurring compounds derived from fat, particularly short chain fatty acids in milk (78) but can be produced from a range of different sources and, apparently, also directly transferred from feed, as evident in this study. Lactones are typically important odour VOC as they have relatively low odour thresholds in milk (79).

## Conclusions

Overall, this study has confirmed that the bovine diet influences the VOC profile of raw milk in relation to its abundance rather than its presence or absence. However, some of the VOC trends in this study did not match those found previously in relation to their abundance and specific diets. Many factors can influence VOC composition in milk through production and analysis; thus, it is difficult to directly compare such studies. DI-HiSorb proved to be a very effective VOC extraction method, as evident by the high number of VOC of different chemical classes extracted and identified in all of these samples (including 99 in the raw milk), and by the fact that panellists could detect so many aromas by GC-O from this extraction method. This study has highlighted that 33 VOC were present in each feed, rumen, and milk sample, thus, eluding to the fact that these are likely transferred directly from the diet into the raw milk. As previously stated, the volatile profile of these raw milks is not that different in terms of content based on diet, but, rather, some significant differences in abundance are evident due to diet, with five VOC significantly higher (*p* < 0.05) in raw GRS milk and ten significantly higher in raw TMR milk. However, despite the fact that only 13 of 99 VOC were significantly different in terms of abundance, the odours of both milk were quite different based on diet as evaluated by olfactometry using OI and AEDA. This is due to the fact that the odour activity of each VOC is based on abundance and an odour threshold, and not abundance alone. The OI of the raw TMR milk (66.2) was greater than that for raw GRS milk (61.2), reflecting the greater abundance of odour activities in the raw TMR milk likely due to its more complex composition as many odour-active VOC appeared to be directly transferred from the diet. Seventeen out of 44 odour activities detected differed between both sample types, with the main characteristic aromas for raw TMR milk deriving from the increased diversity of the TMR diet, which is likely to have been created or enhanced during TMR feed production as many are either derived from Maillard reactions or influenced by heat.

In summary, the following aromas were most associated with both raw GRS and TMR milk: roasted, toasted, bready, potato, popcorn (2-pentyl furan); cheesy, dairy, buttery (butanoic acid); sweet, herbal, fruity, spicey (phenylethyl alcohol); smokey, barnyard, animal, roasted, toasted, cooked potato (2,5-dimethylpyrazine/2,3-dimethylpyrazine); cheesy, sour, sour milk, dairy, nutty, bready, baked, roasted (furfural); sweet, caramel, herbal, fruity, cherry (benzaldehyde/γ-butyrolactone/ethyl hexanoate); green, fresh, earthy, mushroom (1-octen-3-ol; tentative identification); apple, pungent, smokey, burnt, eggy (UNC 4), and fresh, herbal, sweet (UNC 5). Therefore, these odours and associated VOCs have the greatest influence in relation to the sensory character of the milk independent of the diets used in this study. The overall impact of some of these varied between the raw milks based on diet. Several aromas: caramel, sweet, cotton candy (maltol); fishy, cabbage (methanethiol), sweet floral (UNC 6), fresh, sweet, caramel, butterscotch, biscuit, baked (2,3-butanedione), cooked potato, roasty, musty (UNC 1), barbeque, caramel, tobacco, toasted, toffee (γ-hexalactone), toffee, fruity, sweet, caramel (methyl-2-furoate), and vinegar (acetic acid) were much more significant for raw GRS milk than raw TMR milk. Similarly, for raw TMR milk, the following aromas were of greatest impact: smokey, barnyard, animal, roasted, toasted, cooked potato (2,5-dimethylpyrazine/2,3-dimethylpyrazine), barnyard, pungent, animal, solvent (p-cresol/2-pyrrolidone), buttery, animal, barnyard, nutty, bready (3-methylbutanoic acid/2-methylbutanoic acid/heptanal), and smokey, animal, burnt milk [3-/4-ethylphenol (tentative identification)/benzoic acid]. This is also the first time that so many VOCs potentially coming directly from the diet have been shown to influence the aroma of the resultant raw milks. This study clearly highlights the significance of the direct transfer of VOC into raw milk from the diet, the impact of diet, and the potential of DI-HiSorb to extract VOC in these feed, rumen, and raw milk samples.

## Data Availability Statement

The original contributions presented in the study are included in the article/Supplementary Material, further inquiries can be directed to the corresponding author.

## Ethics Statement

Teagasc, has both an Animal Welfare Body and Animal Ethics Committee. The Animal Welfare Body is a legal requirement of Article 26 of Directive 2010/63/EU and Regulation 50 of S.I. No. 543 of 2012. The Health Products Regulatory Authority provided project authorisation. Written informed consent was obtained from the owners for the participation of their animals in this study.

## Author Contributions

HC, KK, and DH designed the research study. HC carried out the experimental work, data collection and analysis, statistical analysis, and drafted the manuscript. EF carried out experimental work. KK, MO'S, DH, and JK reviewed the manuscript. All authors approved the final version of the manuscript.

## Funding

HC is in receipt of the Teagasc Walsh Scholarship (Reference No. 2016071). Funding bodies did not interfere in the design of the study and collection, analysis, and interpretation of data nor in writing the manuscript.

## Conflict of Interest

The authors declare that the research was conducted in the absence of any commercial or financial relationships that could be construed as a potential conflict of interest.

## Publisher's Note

All claims expressed in this article are solely those of the authors and do not necessarily represent those of their affiliated organizations, or those of the publisher, the editors and the reviewers. Any product that may be evaluated in this article, or claim that may be made by its manufacturer, is not guaranteed or endorsed by the publisher.

## References

[1] O'Callaghan T Hennessy D McAuliffe S Kilcawley K O'Donovan M Dillon P. Effect of pasture versus indoor feeding systems on raw milk composition and quality over an entire lactation. J Dairy Sci. (2016) 99:9424–40. 10.3168/jds.2016-10985 27720161

[2] Faccia M. The flavor of dairy products from grass-fed cows. Foods. (2020) 9:1188. 10.3390/foods9091188 32867231 PMC7555911

[3] Salum P Erbay Z Kelebek H Selli S. Optimization of headspace solid-phase microextraction with different fibers for the analysis of volatile compounds of white-brined cheese by using response surface methodology. Food Anal Methods. (2017) 10:1956–64. 10.1007/s12161-016-0774-1

[4] Kilcawley KN Faulkner H Clarke HJ O'Sullivan MG Kerry JP. Factors influencing the flavour of bovine milk and cheese from grass based versus non-grass based milk production systems. Foods. (2018) 7:37. 10.3390/foods7030037 29534042 PMC5867552

[5] Clarke HJ Griffin C Rai DK O'Callaghan TF O'Sullivan MG Kerry JP. Dietary compounds influencing the sensorial, volatile and phytochemical properties of bovine milk. Molecules. (2020) 25:26. 10.3390/molecules25010026 31861730 PMC6983252

[6] Faulkner H O'Callaghan TF McAuliffe S Hennessy D Stanton C O'Sullivan MG. Effect of different forage types on the volatile and sensory properties of bovine milk. J Dairy Sci. (2018) 101:1034–47. 10.3168/jds.2017-13141 29224876

[7] Bugaud C Buchin S Coulon J-B Hauwuy A Dupont D. Influence of the nature of alpine pastures on plasmin activity, fatty acid and volatile compound composition of milk. Lait. (2001) 81:401–14. 10.1051/lait:2001140

[8] Addis M Pinna G Molle G Fiori M Spada S Decandia M. The inclusion of a daisy plant (Chrysanthemum coronarium) in dairy sheep diet: 2. effect on the volatile fraction of milk and cheese. Livest Sci. (2006) 101:68–80. 10.1016/j.livprodsci.2005.09.009

[9] Delahunty CM Eyres G Dufour JP. Gas chromatography-olfactometry. J Sep Sci. (2006) 29:2107–25. 10.1002/jssc.200500509 17069240

[10] Friedrich JE Acree TE. Gas chromatography olfactometry (GC/O) of dairy products. Int Dairy J. (1998) 8:235–41. 10.1016/S0958-6946(98)80002-225405703

[11] Moio L Etievant P Langlois D Dekimpe J Addeo F. Detection of powerful odorants in heated milk by use of extract dilution sniffing analysis. J Dairy Res. (1994) 61:385–94. 10.1017/S0022029900030806

[12] Moio L Langlois D Etievant P Addeo F. Powerful odorants in bovine, ovine, caprine and water buffalo milk determined by means of gas chromatography–olfactometry. J Dairy Res. (1993) 60:215–22. 10.1017/S0022029900027527

[13] Bendall JG. Aroma compounds of fresh milk from New Zealand cows fed different diets. J Agric Food Chem. (2001) 49:4825–32. 10.1021/jf010334n 11600029

[14] Mounchili A Wichtel J Bosset J Dohoo IR Imhof M Altieri D. HS-SPME gas chromatographic characterization of volatile compounds in milk tainted with off-flavour. Int Dairy J. (2005) 15:1203–15. 10.1016/j.idairyj.2004.11.018

[15] Ai N-S Liu H-L Wang J Zhang X-M Zhang H-J Chen H-T. Triple-channel comparative analysis of volatile flavour composition in raw whole and skim milk via electronic nose, GC-MS and GC-O. Anal Methods. (2015) 7:4278–84. 10.1039/C4AY02751E

[16] Mondello L Costa R Tranchida PQ Chiofalo B Zumbo A Dugo P. Determination of flavor components in Sicilian goat cheese by automated HS-SPME-GC. Flavour Fragr J. (2005) 20:659–65. 10.1002/ffj.1529

[17] Bertuzzi AS McSweeney PL Rea MC Kilcawley KN. Detection of volatile compounds of cheese and their contribution to the flavor profile of surface-ripened cheese. Compr Rev Food Sci Food Saf. (2018) 17:371–90. 10.1111/1541-4337.12332 33350078

[18] Thomsen M Gourrat K Thomas-Danguin T Guichard E. Multivariate approach to reveal relationships between sensory perception of cheeses and aroma profile obtained with different extraction methods. Food Res Int. (2014) 62:561–71. 10.1016/j.foodres.2014.03.068

[19] Wang Y Zhao J Xu F Wu X Hu W Chang Y. GC-MS, GC-O and OAV analyses of key aroma compounds in Jiaozi Steamed Bread. Grain and Oil Sci Technol. (2020) 3:9–17. 10.1016/j.gaost.2019.11.003

[20] High R Bremer P Kebede B Eyres GT. Comparison of four extraction techniques for the evaluation of volatile compounds in spray-dried New Zealand sheep milk. Molecules. (2019) 24:1917. 10.3390/molecules24101917 31109044 PMC6571582

[21] Cheng Z Mannion DT O'Sullivan MG Miao S Kerry JP Kilcawley KN. Comparison of automated extraction techniques for volatile analysis of whole milk powder. Foods. (2021) 10:2061. 10.3390/foods10092061 34574176 PMC8467882

[22] Vilar EG Ouyang H O'Sullivan MG Kerry JP Hamill RM O'Grady MN. Effect of salt reduction and inclusion of 1% edible seaweeds on the chemical, sensory and volatile component profile of reformulated frankfurters. Meat Sci. (2020) 161:108001. 10.1016/j.meatsci.2019.108001 31756515

[23] Vilar EG O'Sullivan MG Kerry JP Kilcawley KN. A chemometric approach to characterize the aroma of selected brown and red edible seaweeds/extracts. J Sci Food Agric. (2020) 101:1228–38. 10.1002/jsfa.10735 32790090

[24] Garvey E O'Sullivan M Kerry J Milner L Gallagher E Kilcawley K. Characterising the sensory quality and volatile aroma profile of clean-label sucrose reduced sponge cakes. Food Chem. (2020) 128124. 10.1016/j.foodchem.2020.128124 33127226

[25] Feng Y Cai Y Sun-Waterhouse D Cui C Su G Lin L. Approaches of aroma extraction dilution analysis (AEDA) for headspace solid phase microextraction and gas chromatography–olfactometry (HS-SPME–GC–O): Altering sample amount, diluting the sample or adjusting split ratio? Food Chem. (2015) 187:44–52. 10.1016/j.foodchem.2015.03.138 25976996

[26] R Core Team. A language and environment for statistical computing. R., Foundation for Statistical Computing. Vienna, Austria. (2013).

[27] Villeneuve M-P Lebeuf Y Gervais R Tremblay G Vuillemard J Fortin J. Milk volatile organic compounds and fatty acid profile in cows fed timothy as hay, pasture, or silage. J Dairy Sci. (2013) 96:7181–94. 10.3168/jds.2013-6785 24035021

[28] Croissant AE Washburn S Dean L Drake M. Chemical properties and consumer perception of fluid milk from conventional and pasture-based production systems. J Dairy Sci. (2007) 90:4942–53. 10.3168/jds.2007-0456 17954733

[29] Clarke HJ O'Sullivan MG Kerry JP Kilcawley KN. Correlating volatile lipid oxidation compounds with consumer sensory data in dairy based powders during storage. Antioxidants. (2020) 9:338. 10.3390/antiox9040338 32326117 PMC7222397

[30] Li Y Wang W. Formation of oxidized flavor compounds in concentrated milk and distillate during milk concentration. J Dairy Sci. (2016) 99:9647–51. 10.3168/jds.2016-11619 27743658

[31] Coppa M Martin B Pradel P Leotta B Priolo A Vasta V. Effect of a hay-based diet or different upland grazing systems on milk volatile compounds. J Agric Food Chem. (2011) 59:4947–54. 10.1021/jf2005782 21434695

[32] Morgan M Lindsay R Libbey L Pereira R. Identity of additional aroma constituents in milk cultures of Streptococcus lactis var. maltigenes. J Dairy Sci. (1966) 49:15–8. 10.3168/jds.S0022-0302(66)87777-9 5952149

[33] Ueda Y. Effect of pasture intake on the profile of volatile organic compounds in dairy cow milk. Jpn Agric Res Q. (2018) 52:123–9. 10.6090/jarq.52.12326032306

[34] Vazquez-Landaverde PA Torres JA Qian MC. Quantification of trace volatile sulfur compounds in milk by solid-phase microextraction and gas chromatography–pulsed flame photometric detection. J Dairy Sci. (2006) 89:2919–27. 10.3168/jds.S0022-0302(06)72564-4 16840607

[35] Manousi N Zachariadis GA. Determination of volatile compounds in nut-based milk alternative beverages by HS-SPME prior to GC-MS analysis. Molecules. (2019) 24:3091. 10.3390/molecules24173091 31454898 PMC6751504

[36] Beuvier E Buchin S. Raw milk cheeses. In: Cheese: chemistry, physics and microbiology. Fox P Guinee T Cogan T McSweeney P editors. London, UK: Elsevier. p. 319–45. 10.1016/S1874-558X(04)80072-1

[37] Carpino S Mallia S La Terra S Melilli C Licitra G Acree T. Composition and aroma compounds of Ragusano cheese: native pasture and total mixed rations. J Dairy Sci. (2004) 87:816–30. 10.3168/jds.S0022-0302(04)73226-9 15259216

[38] Hodge J Moser HA. Flavor of bread and pastry upon addition of maltol, isomaltol, and galactosylisomaltol. Cereal Chem. (1961) 38:221–8.

[39] Van Boekel M. Effect of heating on Maillard reactions in milk. Food Chem. (1998) 62:403–14. 10.1016/S0308-8146(98)00075-2

[40] Jo Y Benoist D Barbano D Drake M. Flavor and flavor chemistry differences among milks processed by high-temperature, short-time pasteurization or ultra-pasteurization. J Dairy Sci. (2018) 101:3812–28. 10.3168/jds.2017-14071 29501345

[41] Kilic M Lindsay R. Distribution of conjugates of alkylphenols in milk from different ruminant species. J Dairy Sci. (2005) 88:7–12. 10.3168/jds.S0022-0302(05)72656-4 15591361

[42] Batterham T Hart N Lamberton J Braden A. Metabolism of oestrogenic isoflavones in sheep. Nature. (1965) 206:509. 10.1038/206509a0 5831840

[43] Braden A Hart N Lamberton J. Oestrogenic activity and metabolism of certain isoflavones in sheep. Aust J Agric Res. (1967) 18:355–348. 10.1071/AR9670355

[44] Czerny M Brueckner R Kirchhoff E Schmitt R Buettner A. The influence of molecular structure on odor qualities and odor detection thresholds of volatile alkylated phenols. Chem Senses. (2011) 36:539–53. 10.1093/chemse/bjr009 21441367

[45] Kilcawley K. Milk/Dairy. In: Bordiga M Nollet LM. Food Aroma Evolution. London L, UK: CRC Press. p 465–85. (2019). 10.1201/9780429441837-23

[46] Calvo MM de la Hoz L. Flavour of heated milks. A review. Int Dairy. (1992) 2:69–81. 10.1016/0958-6946(92)90001-3

[47] Alewijn M Smit B Sliwinski E Wouters J. The formation mechanism of lactones in Gouda *cheese*.Int Dairy. (2007) 17:59–66. 10.1016/j.idairyj.2006.01.002

[48] Devos M Patte F Rouault J Laffort P Gemert LJ. Standardized Human Olfactory Thresholds. Press I. editor. Oxford, UK: IRL Press. (1990).

[49] Leffingwell Associates. Odor Thresholds. (2020). Available online at: http://www.leffingwell.com/odorthre.htm (accessed 03 Dec, 2020).

[50] New Jersey Department of Health. Hazardous Substance Fact Sheet Acetic Acid. (2016). Available online at: https://www.nj.gov/health/eoh/rtkweb/documents/fs/0004.pdf (accessed December 03, 2020).

[51] Leonardos G Kendall D Barnard N. Odor threshold determination of 53 odorant chemicals. J Environ Conserv Eng. (1974) 3:579–85. 10.5956/jriet.3.57915161224

[52] Franco M Peinado RA Medina M Moreno J. Off-vine grape drying effect on volatile compounds and aromatic series in must from Pedro Ximénez grape variety. J Agric Food Chem. (2004) 52:3905–10. 10.1021/jf0354949 15186115

[53] US National Library of Medicine National Center for Biotechnology Information. (2016). Available online at: https://www.ncbi.nlm.nih.gov/ (accessed 26 Oct, 2021).

[54] Burdock G. Fenaroli's Handbook of Flavor Ingredients., Fourth ed. London, U. K.: C. R. C. Press. (2001).

[55] Poisson L Schieberle P. Characterization of the key aroma compounds in an American Bourbon whisky by quantitative measurements, aroma recombination, omission studies. J Agric Food Chem. (2008) 56:5820–6. 10.1021/jf800383v 18582086

[56] Guo S Jom KN Ge Y. Influence of Roasting Condition on Flavor Profile of Sunflower Seeds: A flavoromics approach. Sci Rep. (2019) 9:1–10. 10.1038/s41598-019-47811-3 31383910 PMC6683164

[57] Cliff M Stanich K Trujillo JM Toivonen P Forney CF. Determination and prediction of odor thresholds for odor active volatiles in a neutral apple juice matrix. J Food Qual. (2011) 34:177–86. 10.1111/j.1745-4557.2011.00383.x

[58] Liu J Zhao W Li S Zhang A Zhang Y Liu S. Characterization of the key aroma compounds in proso millet wine using headspace solid-phase microextraction and gas chromatography-mass spectrometry. Molecules. (2018) 23:462. 10.3390/molecules23020462 29461466 PMC6017027

[59] Peinado RA Moreno J Bueno JE Moreno JA Mauricio JC. Comparative study of aromatic compounds in two young white wines subjected to pre-fermentative cryomaceration. Food Chem. (2004) 84:585–90. 10.1016/S0308-8146(03)00282-6

[60] Czerny M Christlbauer M Christlbauer M Fischer A Granvogl M Hammer M. Re-investigation on odour thresholds of key food aroma compounds and development of an aroma language based on odour qualities of defined aqueous odorant solutions. Eur Food Res Technol. (2008) 228:265–73. 10.1007/s00217-008-0931-x

[61] Dietz F Traud J. Odor and taste threshold concentrations of phenolic compounds. GWF, Wasser/Abwasser. (1978) 119:318–25.23578617

[62] Siek T Albin I Sather L Lindsay R. Comparison of flavor thresholds of aliphatic lactones with those of fatty acids, esters, aldehydes, alcohols, and ketones. J Dairy Sci. (1971) 54:1–4. 10.3168/jds.S0022-0302(71)85770-3

[63] Stewart A Grandison A Fagan C Ryan A Festring D Parker JK. Changes in the volatile profile of skim milk powder prepared under different processing conditions and the effect on the volatile flavor profile of model white chocolate. J Dairy Sci. (2018) 101:8822–36. 10.3168/jds.2018-14414 30122413

[64] Riuzzi G Tata A Massaro A Bisutti V Lanza I Contiero B. Authentication of forage-based milk by mid-level data fusion of (+/–) DART-HRMS signatures. Int Dairy J. (2021) 112:104859. 10.1016/j.idairyj.2020.104859

[65] Yelliantty Y Kartasamita RE Surantaatmadja SI Rukayadi Y. Identification of chemical constituents from fruit of Antidesma bunius by GC-MS and HPLC-DAD-ESI-MS. Food Sci Technol. (2021). 10.1590/fst.61320

[66] Castellani F Bernardi N Vitali A Marone E Grotta L Martino G. Proteolytic volatile compounds in milk and cheese of cows fed dried olive pomace supplementation. J Anim Feed Sci. (2018) 27:361–5. 10.22358/jafs/99600/2018

[67] Dutta S De S Saha B Alam MI. Advances in conversion of hemicellulosic biomass to furfural and upgrading to biofuels. Catal Sci Technol. (2012) 2:2025–2036. 10.1039/c2cy20235b

[68] Grewal J Khare S. 2-Pyrrolidone synthesis from γ-aminobutyric acid produced by Lactobacillus brevis under solid-state fermentation utilizing toxic deoiled cottonseed cake. Bioprocess Biosyst Eng. (2017) 40:145–52. 10.1007/s00449-016-1683-9 27646908

[69] Clarke H Griffin C Hennessy D O'Callaghan T O'Sullivan M Kerry J. Effect of bovine feeding system (pasture or concentrate) on the oxidative and sensory shelf life of whole milk powder. J Dairy Sci. (2021) 104:10654–68. 10.3168/jds.2021-20299 34275630

[70] Sieber R Bütikofer U Bosset J. Benzoic acid as a natural compound in cultured dairy products and cheese. Int Dairy J. (1995) 5:227–46. 10.1016/0958-6946(94)00005-A

[71] Bosset J Bütikofer U Sieber R. Phenylalanine breakdown-another pathway of natural production of benzoic acid in smeared cheese. Schweizerische Milchwirtschaftliche Forschung. (1990) 19:46–50.

[72] Mackle T Dwyer D Bauman D. Effects of branched-chain amino acids and sodium caseinate on milk protein concentration and yield from dairy cows. J Dairy Sci. (1999) 82:161–71. 10.3168/jds.S0022-0302(99)75220-3 10022018

[73] Toso B Procida G Stefanon B. Determination of volatile compounds in cows' milk using headspace GC-MS. J Dairy Res. (2002) 69:569–77. 10.1017/S0022029902005782 12463694

[74] Tawfik MM Yamato KT Kohchi T Koeduka T Matsui K. n-Hexanal and (Z)-3-hexenal are generated from arachidonic acid and linolenic acid by a lipoxygenase in *Marchantia polymorpha* L. Biosci Biotechnol Biochem. (2017) 81:1148–55. 10.1080/09168451.2017.1285688 28162041

[75] García-Martínez M Márquez-Ruiz G Fontecha J Gordon M. Volatile oxidation compounds in a conjugated linoleic acid-rich oil. Food Chem. (2009) 113:926–31. 10.1016/j.foodchem.2008.08.020

[76] Bergamaschi M Bittante G. From milk to cheese: Evolution of flavor fingerprint of milk, cream, curd, whey, ricotta, scotta, and ripened cheese obtained during summer Alpine pasture. J Dairy Sci. (2018) 101:3918–34. 10.3168/jds.2017-13573 29454692

[77] Hoffmann A Heiden A. Determination of flavor and off flavor compounds in dairy products using stir bar sorptive extraction (SBSE) and thermal desorption GC/MSD/PFPD. 23rd international symposium on Capillary. Chromatography. (2000).

[78] Dimick P Harner J. Effect of environmental factors on lactone potential in bovine milk fat. J Dairy Sci. (1968) 51:22–7. 10.3168/jds.S0022-0302(68)86912-7

[79] Patton S. Browning and associated changes in milk and its products: A review. J Dairy Sci. (1955) 38:457–78. 10.3168/jds.S0022-0302(55)95000-1

