# Supplementary material for: The Influence of Pasture and Non-pasture-Based Feeding Systems on the Aroma of Raw Bovine Milk
Source: Front Nutr. 2022 Mar 10;9:841454. doi: 10.3389/fnut.2022.841454 (PMC8960744; doi:10.3389/fnut.2022.841454)
Supplement: Supplementary file 1 [file Table_1.DOCX]

Supplementary Table 1: Volatile compounds identified in grass and total mixed ration (TMR) raw milk (n=3), feed (n=3), rumen fluid (n=3) and rumen blended (n=3) samples via HiSorb gas chromatography mass spectrometry.

| **Compound** | **CAS no.** | **RI** | **IM** | **Grass feed** | **TMR feed** | **Grass RF** | **TMR RF** | **Grass RB** | **TMR RB** | **Raw GRS milk** | **Raw TMR milk** | **Occurrence** |
| --- | --- | --- | --- | --- | --- | --- | --- | --- | --- | --- | --- | --- |
| **Acids** | | | | | | | | | | | | |
| Formic acid | 64-18-6 | 605.8 | MS | ND | ND | ND | ND | ND | ND | 8.53x10^4^ | 6.58x10^4^ | M |
| Acetic acid | 64-19-7 | 662.8 | MS, IHL, LRI | 3.72x10^6^ | 2.29x10^7^ | 2.61x10^7^ | 4.58x10^7^ | 7.11x10^7^ | 5.26x10^7^ | 1.96x10^6^ | 2.57x10^6^ | AO |
| Propanoic acid | 79-09-4 | 786 | MS, IHL, LRI | 1.69x10^6^ | 1.16x10^7^ | 1.19x10^7^ | 2.72x10^7^ | 3.16x10^7^ | 2.70x10^7^ | 2.49x10^5^ | 2.64x10^5^ | AO |
| 2-Methylpropanoic acid | 79-31-2 | 841.8 | MS | ND | ND | ND | ND | ND | ND | 1.92x10^4^ | 1.44x10^4^ | M |
| 2-Methyl-2-propenoic acid | 79-41-4 | 867.1 | MS, LRI | ND | ND | ND | ND | ND | ND | 3.78x10^4^ | 5.37x10^4^ | M |
| Butanoic acid | 107-92-6 | 914.4 | MS, LRI | 3.68x10^6^ | 9.57x10^7^ | 3.03x10^7^ | 8.33x10^7^ | 1.03x10^8^ | 1.79x10^8^ | 7.93x10^5^ | 1.10x10^6^ | AO |
| 2-Methylbutanoic acid | 116-53-0 | 841.8 | MS, IHL, LRI | ND | ND | ND | ND | ND | ND | 2.07x10^4^ | 1.13x10^5^ | MO |
| 3-Methylbutanoic acid | 503-74-2 | 914.5 | MS | ND | 2.61x10^6^ | 3.35x10^6^ | 1.01x10^7^ | 1.24x10^7^ | 1.37x10^7^ | 0.00x00 | 4.35x10^4^ | - |
| Pentanoic acid | 109-52-4 | 973.9 | MS | 8.40x10^5^ | 1.54x10^7^ | 8.15x10^6^ | 5.50x10^7^ | 2.20x10^7^ | 8.47x10^7^ | 1.60x10^5^ | 3.77x10^5^ | - |
| Hexanoic acid | 142-62-1 | 1069.7 | MS, IHL, LRI | 1.01x10^6^ | 8.67x10^7^ | 9.27x10^6^ | 1.14x10^8^ | 2.05x10^7^ | 1.57x10^8^ | 7.81x10^5^ | 1.67x10^6^ | A |
| Heptanoic acid | 111-14-8 | 1164.1 | MS, IHL | ND | ND | ND | ND | ND | ND | 1.82x10^5^ | 3.53x10^5^ | - |
| Octanoic acid | 124-07-2 | 1261.9 | MS | 7.32x10^5^ | 3.28x10^7^ | 1.44x10^6^ | 1.16x10^7^ | 2.05x10^6^ | 1.22x10^7^ | 1.41x10^6^ | 3.45x10^6^ | O |
| Benzoic acid | 65-85-0 | 1285.2 | MS | ND | ND | ND | ND | ND | ND | 2.35x10^5^ | 3.40x10^4^ | MO |
| Nonanoic acid | 112-05-0 | 1353.8 | MS, IHL | ND | ND | ND | ND | ND | ND | 3.98x10^5^ | 6.88x10^5^ | M |
| Decanoic acid | 334-48-5 | 1439.0 | MS, LRI | 1.77x10^6^ | 1.77x10^7^ | 4.39x10^6^ | 4.65x10^6^ | 3.99x10^6^ | 6.04x10^6^ | 2.97x10^6^ | 6.71x10^6^ | - |
| Decanoic acid | 334-48-5 | 1452.3 | MS, IHL | 1.77x10^6^ | 1.77x10^7^ | 4.39x10^6^ | 4.65x10^6^ | 3.99x10^6^ | 6.04x10^6^ | 2.97x10^6^ | 6.71x10^6^ | - |
| Hydrocinnamic acid | 501-52-0 | 1460 | MS | ND | ND | ND | ND | ND | ND | 8.27x10^4^ | 2.89x10^4^ | MO |
| Undecanoic acid | 112-37-8 | 1544 | MS, LRI | ND | ND | 1.67x10^4^ | 5.44x10^5^ | ND | ND | 8.26x10^4^ | 1.45x10^5^ | - |
| Dodecanoic acid | 143-07-7 | 1640.2 | MS | 6.15x10^5^ | 7.63x10^6^ | 3.36x10^6^ | 1.35x10^7^ | 2.39x10^6^ | 5.12x10^6^ | 2.02x10^6^ | 1.94x10^6^ | AO |
| Tetradecanoic acid | 544-63-8 | 1839.7 | MS | ND | ND | ND | ND | ND | ND | 4.39x10^5^ | 2.91x10^5^ | - |
| **Alcohols** | | | | | | | | | | | | |
| Ethanol | 64-17-5 | 504.3 | MS, IHL, LRI | 8.61x10^5^ | 5.18x10^6^ | 3.79x10^6^ | 3.52x10^6^ | 2.21x10^6^ | 3.30x10^6^ | 3.51x10^6^ | 2.74x10^6^ | A |
| Isopropyl alcohol | 67-63-0 | 542.2 | MS | 4.70x10^5^ | 6.38x10^5^ | 1.17x10^6^ | 9.04x10^5^ | 5.50x10^5^ | 1.02x10^6^ | ND | ND | - |
| Phenylethyl Alcohol | 60-12-8 | 542.2 | MS | 1.31x10^6^ | 2.76x10^7^ | 2.92x10^6^ | 6.44x10^6^ | 1.80x10^6^ | 6.88x10^6^ | 1.09x10^4^ | 1.42x10^4^ | AO |
| 1-Propanol | 71-23-8 | 611.3 | MS, LRI | 3.99x10^3^ | 8.75x10^5^ | 2.19x10^5^ | 5.58x10^5^ | 1.26x10^6^ | 1.79x10^6^ | ND | ND | - |
| 2-Butanol | 78-92-2 | 647.4 | MS, LRI | 1.87x10^4^ | 6.48x10^6^ | 7.19x10^5^ | 9.02x10^6^ | 1.14x10^6^ | 1.65x10^7^ | ND | ND | - |
| 1-Propanol, 2-methyl- | 78-83-1 | 678.2 | MS, LRI | 2.87x10^4^ | 8.43x10^5^ | 1.87x10^5^ | 3.33x10^5^ | 2.35x10^5^ | 5.23x10^5^ | ND | ND | - |
| 1-Butanol | 71-36-3 | 688.9 | MS, IHL | 1.10x10^5^ | 5.37x10^6^ | 2.40x10^6^ | 5.90x10^6^ | 5.69x10^6^ | 9.15x10^6^ | 9.70x10^3^ | ND | A |
| 2-Methyl-1-butanol | 137-32-6 | 787.1 | MS, LRI | 3.01x10^5^ | 3.14x10^6^ | 6.94x10^5^ | 1.80x10^6^ | 4.87x10^5^ | 1.78x10^6^ | 2.20x10^5^ | 1.19x10^4^ | A |
| 4-Methyl-2-pentanol | 108-11-2 | 796.4 | MS, IHL, LRI | ND | ND | ND | ND | ND | ND | 7.85x10^3^ | 3.54x10^3^ | M |
| 3-Methyl-1-butanol | 123-51-3 | 803.2 | MS, LRI | 7.59x10^5^ | 1.53x10^7^ | 1.84x10^6^ | 4.76x10^6^ | 2.98x10^6^ | 8.94x10^6^ | ND | ND | - |
| 1-Pentanol | 71-41-0 | 810.6 | MS, IHL, LRI | 9.06x10^5^ | 1.53x10^7^ | 2.16x10^6^ | 6.53x10^6^ | 2.98x10^6^ | 8.94x10^6^ | 1.07x10^5^ | 1.95x10^4^ | AO |
| 1-Hexanol | 111-27-3 | 915.4 | MS, LRI | 7.57x10^6^ | 7.47x10^6^ | 2.44x10^6^ | 1.95x10^7^ | 1.52x10^6^ | 1.93x10^7^ | 1.88x10^4^ | 3.75x10^3^ | A |
| 2-Furanmethanol | 98-00-0 | 929.5 | MS, LRI | ND | ND | ND | ND | ND | ND | 5.21x10^6^ | 6.80x10^6^ | MO |
| Ethanol, 2-butoxy- | 111-76-2 | 954.5 | MS | 3.47x10^4^ | ND | ND | ND | ND | ND | 3.78x10^4^ | 9.83x10^4^ | - |
| 3-Furanmethanol | 4412-91-3 | 1046.4 | MS | ND | ND | ND | ND | ND | ND | 1.81x10^5^ | 2.33x10^5^ | M |
| Benzyl alcohol | 100-51-6 | 1122 | MS | ND | 4.21x10^6^ | ND | ND | 2.72x10^5^ | 2.99x10^5^ | ND | ND | - |
| 1-Octen-3-ol | 3391-86-4 | 1028 | MS, IHL, LRI | ND | ND | ND | ND | ND | ND | 5.18x10^4^ | ND | MO |
| 3-Methyl-1-hexyn-3-ol | 4339-05-3 | 1046.4 | MS | ND | ND | ND | ND | ND | ND | 9.68x10^2^ | 6.44x10^2^ | M |
| Dihydroxyacetone | 96-26-4 | 1040.6 | MS, LRI | ND | ND | ND | ND | ND | ND | 2.76x10^4^ | 1.35x10^4^ | M |
| Ethanol, 2-(2-ethoxyethoxy)- | 111-90-0 | 1061.2 | MS | 4.11x10^4^ | ND | 7.33x10^4^ | 1.02x10^5^ | ND | ND | ND | ND | - |
| 1-Hexanol, 2-ethyl- | 104-76-7 | 1076.4 | MS, LRI | 1.61x10^6^ | 4.84x10^5^ | 1.40x10^6^ | 7.40x10^5^ | 2.19x10^5^ | 2.62x10^5^ | 9.46x10^4^ | 7.65x10^4^ | AO |
| 1-Octanol | 111-87-5 | 1120.1 | MS, IHL | 2.44x10^5^ | 2.85x106 | 1.14x10^5^ | 2.72x10^5^ | 1.92x10^5^ | 9.23x10^5^ | 7.39x10^4^ | 5.37x10^4^ | AO |
| 2-Phenoxyethanol | 122-99-6 | 1320.4 | MS, IHL, LRI | 3.71x10^5^ | ND | 9.99x10^4^ | ND | ND | ND | 2.94x10^5^ | 1.55x10^5^ | MO |
| 1-Dodecanol | 112-53-8 | 1523.3 | MS, LRI | 2.69x10^5^ | 1.02x10^5^ | 8.39x10^5^ | 1.44x10^6^ | 1.99x10^5^ | 4.43x10^5^ | 1.35x10^5^ | 1.05x10^5^ | A |
| Tetradecanol | 112-72-1 | 1724.2 | MS | 2.36x10^4^ | ND | 3.60x10^4^ | 6.73x10^4^ | ND | ND | 1.49x10^5^ | 1.78x10^5^ | AO |
| **Aldehydes** | | | | | | | | | | | | |
| Acetaldehyde | 75-07-0 | 451.7 | MS | 1.06x10^6^ | 1.63x10^6^ | 2.97x10^6^ | 2.94x10^6^ | 3.30x10^6^ | 2.75x10^6^ | 5.50x10^6^ | 4.97x10^6^ | A |
| 2-Propenal | 107-02-8 | 524.3 | MS | 2.26x10^5^ | 2.63x10^5^ | 2.24x10^5^ | 3.26x10^5^ | 2.72x10^5^ | 2.44x10^5^ | ND | ND | - |
| Propanal | 123-38-6 | 528.2 | MS | 2.34x10^5^ | 1.23x10^5^ | 7.99x10^4^ | 7.19x10^4^ | 1.41x10^5^ | 1.09x10^5^ | ND | ND | - |
| 2-Methyl-propanal | 78-84-2 | 594.4 | MS | 2.50x10^5^ | 3.62x10^5^ | 1.61x10^5^ | 1.65x10^5^ | 3.20x10^5^ | 2.61x10^5^ | ND | 4.93x10^5^ | O |
| Butanal | 123-72-8 | 633.4 | MS | 8.38x10^5^ | 8.50x10^6^ | 7.57x10^5^ | 9.42x10^5^ | 1.02x10^6^ | 1.22x10^7^ | ND | ND | - |
| 3-Methy-l-butanal | 590-86-3 | 650.4 | MS, IHL, LRI | 1.15x10^6^ | 8.85x10^5^ | 2.16x10^5^ | 2.27x10^5^ | 5.16x10^5^ | 3.43x10^5^ | 2.43x10^4^ | 2.24x10^4^ | A |
| 2-Methy-l-butanal | 96-17-3 | 700.4 | MS | 5.83x10^6^ | 2.29x10^5^ | ND | ND | 6.89x10^5^ | 5.44x10^5^ | ND | ND | - |
| Hexanal | 66-25-1 | 828.9 | MS, IHL | 2.10x10^6^ | 2.15x10^5^ | ND | ND | ND | ND | 2.21x10^5^ | 1.85x10^5^ | O |
| Furfural | 98-01-1 | 899.3 | MS | 1.59x10^5^ | 1.30x10^6^ | ND | ND | 4.50x10^5^ | 6.50x10^5^ | 1.09x10^6^ | 1.31x10^6^ | O |
| (E)-2-Hexenal | 6728-26-3 | 901.2 | MS | ND | ND | ND | ND | ND | ND | 1.35x10^4^ | 5.05x10^3^ | M |
| Heptanal | 111-71-7 | 943.1 | MS | 8.52x10^5^ | 7.74x10^5^ | 6.81x10^5^ | 7.17x10^5^ | 3.01x10^5^ | 4.02x10^5^ | 2.13x10^5^ | 1.60x10^5^ | AO |
| Benzaldehyde | 100-52-7 | 1031.6 | MS | 2.02x10^6^ | 3.67x10^6^ | 3.84x10^6^ | 3.11x10^6^ | 2.10x10^6^ | 1.66x10^6^ | 2.19x10^5^ | 2.03x10^5^ | AO |
| 5-methyl furfural | 620-02-0 | 1040.6 | MS | ND | ND | ND | ND | ND | ND | 1.73x10^5^ | 2.07x10^5^ | M |
| (E,E)-2,4-Heptadienal | 4313-03-5 | 1074.2 | MS, LRI | ND | ND | ND | ND | ND | ND | 2.56x10^4^ | 1.39x10^3^ | M |
| Benzeneacetaldehyde | 122-78-1 | 1108.5 | MS, IHL, LRI | 3.05x10^6^ | ND | 2.92x10^5^ | ND | 2.56x10^5^ | 9.18x10^3^ | 2.76x10^4^ | 3.94x10^4^ | O |
| Nonanal | 124-19-6 | 1147.6 | MS, IHL, LRI | 3.70x10^6^ | 4.10x10^6^ | 3.45x10^6^ | 3.04x10^6^ | 1.81x10^6^ | 1.38x10^6^ | 1.11x10^6^ | 7.50x10^5^ | O |
| Decanal | 112-31-2 | 1252.6 | MS, IHL, LRI | 1.12x10^6^ | 9.88x10^5^ | 1.41x10^6^ | 1.12x10^6^ | 6.27x10^5^ | 5.75x10^5^ | 4.16x10^5^ | 3.02x10^5^ | AO |
| Dodecanal | 112-54-9 | 1457.2 | MS, IHL | 9.39x10^4^ | 7.11x104 | 4.28x10^5^ | 1.12x10^6^ | 2.55x10^4^ | 1.51x10^5^ | 5.68x10^4^ | 0.00x00 | - |
| Tridecanal | 10486-19-8 | 1558.7 | MS, LRI | ND | ND | 1.08x10^5^ | 2.52x10^5^ | ND | ND | 2.28x10^4^ | 8.22x10^3^ | - |
| Undecanal | 112-44-7 | 1357.7 | MS, LRI | ND | ND | 6.62x10^4^ | ND | ND | ND | ND | ND | - |
| Octanal | 124-13-0 | 1046.9 | MS | 5.92x10^5^ | 7.53x10^5^ | 9.14x10^5^ | 4.52x10^5^ | 3.06x10^5^ | 4.69x10^5^ | 3.07x10^5^ | 2.27x10^5^ | A |
| **Esters and Ethers** | | | | | | | | | | | | |
| Ethyl ether | 60-29-7 | 514.2 | MS | ND | ND | ND | ND | 1.88x10^4^ | ND | ND | ND | - |
| Ethyl acetate | 141-78-6 | 641.1 | MS | 3.27x10^5^ | 8.59x10^6^ | 8.47x10^5^ | 6.20x10^6^ | 5.33x10^5^ | 4.81x10^6^ | ND | ND | - |
| Methyl propionate | 922-67-8 | 657.6 | MS | ND | 5.27x10^4^ | ND | 7.01x10^3^ | 4.82x10^5^ | 8.80x10^5^ | ND | ND | - |
| Ethyl propanoate | 105-37-3 | 736.5 | MS | ND | 2.39x10^6^ | 1.99x10^6^ | 1.23x10^7^ | 2.25x10^6^ | 1.04x10^7^ | ND | ND | - |
| Methyl methacrylate | 80-62-6 | 738.4 | MS | 1.53x10^5^ | 6.53x10^4^ | 6.10x10^4^ | 3.89x10^4^ | ND | ND | ND | ND | - |
| n-Propyl acetate | 109-60-4 | 741.7 | MS | ND | 1.17x10^6^ | ND | 6.73x10^6^ | 2.08x10^4^ | 1.51x10^6^ | ND | ND | - |
| Methyl butanoate | 623-42-7 | 749.2 | MS | 1.81x10^4^ | 1.72x10^7^ | 3.59x10^4^ | 4.77x10^4^ | 2.93x10^6^ | 1.20x10^7^ | ND | ND | - |
| Isobutyl acetate | 110-19-0 | 800.3 | MS | ND | 4.22x10^5^ | ND | 1.73x10^5^ | ND | ND | ND | ND | - |
| Ethyl butanoate | 903170-13-8 | 825.1 | MS | 3.71x10^5^ | 1.74x10^8^ | 7.00x10^6^ | 9.21x10^7^ | 1.93x10^6^ | 1.88x10^7^ | ND | ND | - |
| Butyl acetate | 123-86-4 | 834.2 | MS, IHL, LRI | ND | 2.54x10^6^ | 3.01x10^5^ | 9.80x10^6^ | ND | ND | 7.36x10^3^ | 3.51x10^3^ | - |
| Ethyl 2-methylbutanoate | 7452-79-1 | 874.1 | MS | ND | ND | ND | ND | 1.59x10^5^ | 1.39x10^7^ | ND | ND | - |
| Ethyl 3-methylbutanoate | 108-64-5 | 877.4 | MS | ND | ND | 3.20x10^5^ | 3.38x10^6^ | 6.60x10^4^ | 9.53x10^5^ | ND | ND | - |
| Ethylbenzene | 100-41-4 | 891.4 | MS | ND | ND | 1.37x10^5^ | 6.84x10^4^ | 8.33x10^4^ | 2.60x10^4^ | ND | ND | - |
| Ethyl lactate | 97-64-3 | 867.5 | MS | ND | 1.68x10^7^ | ND | ND | ND | ND | ND | ND | - |
| Ethyl 2-methylbutanoate | 7452-79-1 | 873.9 | MS | 2.34x10^4^ | 4.06x10^5^ | 2.91 x10^5^ | 4.36 x10^7^ | ND | ND | ND | ND | - |
| Isoamyl acetate | 123-92-2 | 902.5 | MS | 5.14x10^4^ | 5.61x10^6^ | 2.57x10^5^ | 2.16x10^6^ | 4.75x10^4^ | 4.49x10^5^ | ND | ND | - |
| 2-Methylbutyl acetate | 624-41-9 | 902.7 | MS | ND | 6.38x10^6^ | 4.90x10^3^ | 3.15x10^5^ | ND | 2.47x10^5^ | ND | ND | - |
| 1-Methylpropyl ester butanoic acid | 819-97-6 | 903 | MS | ND | ND | ND | ND | ND | ND | 6.56x10^3^ | ND | M |
| Amyl acetate | 628-63-7 | 915.5 | MS | ND | 5.61x10^6^ | ND | ND | ND | 1.05x10^6^ | ND | ND | - |
| Propyl butyrate | 105-66-8 | 923.2 | MS | 1.09x10^4^ | 3.20x10^7^ | 4.51x10^6^ | 2.12x10^6^ | 1.73x10^5^ | 2.08x10^5^ | ND | ND | - |
| Ethyl pentanoate | 539-82-2 | 925.2 | MS | 2.86x10^5^ | 1.31x10^7^ | ND | ND | ND | ND | ND | ND | - |
| Propanoic acid, butyl ester | 590-01-2 | 934 | MS | ND | 7.23x10^5^ | 3.89x10^6^ | 5.03x10^6^ | 5.27x10^4^ | 5.73x10^5^ | ND | ND | - |
| Pentyl acetate | 628-63-7 | 941.4 | MS | ND | ND | ND | 2.19x10^6^ | ND | ND | ND | ND | - |
| Methyl hexanoate | 106-70-7 | 952.9 | MS | 2.07x10^4^ | 3.61x10^7^ | ND | 2.37x10^5^ | 1.24x10^6^ | 3.15x10^7^ | ND | ND | - |
| Isobutyl butyrate | 539-90-2 | 964.3 | MS | ND | 5.09x10^6^ | 3.52x10^6^ | 1.10x10^7^ | ND | ND | ND | ND | - |
| Propanoic acid, 2-methyl-, 3-hydroxy-2,2,4-trimethylpentyl ester | 77-68-9 | 974.2 | MS | ND | ND | 1.57x10^5^ | 4.69x10^4^ | ND | ND | ND | ND | - |
| Propyl 2-methylbutanoate | 37064-20-3 | 974.2 | MS | ND | 3.05x10^4^ | 4.96x10^5^ | 1.82x10^7^ | 1.37x10^4^ | 2.20x10^6^ | ND | ND | - |
| Amyl propionate | 624-54-4 | 997.9 | MS | ND | 9.48x10^5^ | 1.55x10^5^ | 9.94x10^5^ | ND | 1.13x10^5^ | ND | ND | - |
| Butyl butanoate | 100-52-7 | 1023.6 | MS | ND | 4.91x10^7^ | 1.87x10^6^ | 2.15x10^6^ | 1.54x10^4^ | 1.34x10^5^ | 8.17x10^3^ | 0.00x00 | - |
| Ethyl hexanoate | 123-66-0 | 1026.2 | MS | ND | 1.73x10^8^ | 4.81x10^5^ | 1.97x10^8^ | 1.47x10^5^ | 2.65x10^7^ | 1.28x10^5^ | 1.41x10^4^ | O |
| Hexyl acetate | 142-92-7 | 1042.1 | MS | ND | 8.08x10^5^ | ND | 1.34x10^6^ | ND | ND | ND | ND | - |
| 2-Methylbutyl butanoate | 51115-64-1 | 1082.4 | MS | ND | 3.43x10^7^ | 1.45x10^5^ | 1.43x10^6^ | ND | 2.19x10^5^ | ND | ND | - |
| Isoamyl isobutanoate | 2050-01-3 | 1082.4 | MS | ND | 3.43x10^7^ | 6.09x10^4^ | 1.58x10^6^ | ND | ND | ND | ND | - |
| Propyl hexanoate | 626-77-7 | 1120.2 | MS | ND | 6.64x10^7^ | ND | 2.61x10^7^ | ND | 1.94x10^6^ | ND | ND | - |
| Ethyl heptanoate | 106-30-9 | 1122.8 | MS | ND | ND | ND | 2.46x10^7^ | ND | 2.32x10^6^ | ND | ND | - |
| Isobutyl hexanoate | 105-79-3 | 1156.5 | MS | ND | 2.16x10^7^ | ND | 8.08x10^6^ | ND | ND | ND | ND | - |
| Methyl-2-furoate | 611-13-2 | 1170 | MS | ND | ND | ND | ND | ND | ND | 4.18x10^5^ | 4.88x10^5^ | MO |
| Butyl hexanoate | 626-82-4 | 1217.8 | MS | ND | 2.08x10^7^ | ND | ND | ND | ND | ND | ND | - |
| Ethyl benzoate | 93-89-0 | 1233 | MS | ND | 3.49x10^6^ | 1.01x10^5^ | 4.90x10^5^ | ND | 5.81x10^4^ | ND | ND | - |
| Ethyl octanoate | 106-32-1 | 1225.7 | MS, IHL, LRI | ND | 7.76x10^6^ | 2.18x10^5^ | 2.99x10^6^ | 6.39x10^4^ | 5.12x10^5^ | 8.64x10^4^ | 2.02x10^4^ | O |
| Methyl benzeneacetate | 101-41-7 | 1241.3 | MS | ND | ND | ND | ND | 4.69x10^5^ | 7.98x10^5^ | ND | ND | - |
| Ethyl benzeneacetate | 101-97-3 | 1306.6 | MS | ND | 5.66x10^6^ | ND | 4.65x10^5^ | ND | 1.32x10^5^ | ND | ND | - |
| B-Phenylethyl acetate | 103-45-7 | 1322.1 | MS | ND | 6.06x10^6^ | ND | ND | ND | ND | ND | ND | - |
| Butyl ethyl succinate | 67233-92-5 | 1360 | MS | ND | 9.23x10^4^ | ND | ND | ND | ND | ND | ND | - |
| Ethyl decanoate | 110-38-3 | 1422.5 | MS, IHL, LRI | ND | 6.73x10^5^ | ND | 1.29x10^6^ | ND | 4.83x10^5^ | 9.29x10^4^ | 3.18x10^4^ | - |
| Ethyl dodecanoate | 106-33-2 | 1620 | MS, IHL, LRI | ND | 1.21x10^5^ | ND | ND | ND | 1.69x10^4^ | 1.13x10^4^ | 7.60x10^3^ | - |
| Ethyl hexadecanoate | 628-97-7 | 2029 | MS | ND | 2.85x10^5^ | ND | ND | ND | ND | ND | ND | - |
| **Furans** | | | | | | | | | | | | |
| Furan | 110-00-9 | 518.9 | MS | 9.43x10^4^ | 9.98x10^4^ | 5.03x10^4^ | 8.96x10^4^ | 7.91x10^4^ | 5.25x10^4^ | ND | ND | - |
| 2-Ethylfuran | 3208-16-0 | 718.9 | MS | 1.22x10^6^ | 7.65x10^4^ | ND | ND | ND | ND | ND | ND | - |
| 2-Methylfuran | 79-09-4 | 793.9 | MS | 8.31x10^4^ | 2.11x10^5^ | 6.34x10^4^ | 7.74x10^4^ | 1.29x10^5^ | 7.76x10^4^ | 1.36x10^4^ | 2.25x10^4^ | - |
| 2-n-Butyl furan | 4466-24-4 | 912.3 | MS | ND | ND | 8.72x10^3^ | 3.80x10^3^ | ND | ND | ND | ND | - |
| 2-Pentylfuran | 3777-69-3 | 1008.9 | MS, IHL, LRI | 4.94x10^5^ | 1.25x10^6^ | 1.35x10^6^ | 1.25x10^6^ | 4.47x10^5^ | 5.72x10^5^ | 7.76x10^3^ | 1.31x10^4^ | AO |
| Isomaltol | 3420-59-5 | 1040.6 | MS, LRI | ND | ND | ND | ND | ND | ND | 1.22x10^6^ | 1.10x10^6^ | MO |
| Diethyl succinate | 123-25-1 | 1227.5 |  | ND | 1.26x10^6^ | ND | ND | ND | ND | 2.49x10^3^ | 1.26x10^3^ | - |
| **Hydrocarbons and Benzenes** | | | | | | | | | | | | |
| Benzene | 71-43-2 | 686.9 | MS | 3.15x10^5^ | 6.36x10^5^ | 6.57x10^5^ | 5.20x10^5^ | 1.72x10^5^ | 2.88x10^5^ | ND | ND | - |
| Toluene | 108-88-3 | 773.5 | MS, IHL | 5.71x10^5^ | 5.06x10^5^ | 3.20x10^7^ | 1.97x10^6^ | 7.03x10^6^ | 1.04x10^6^ | 1.14x10^5^ | 1.53x10^4^ | AO |
| p-Xylene | 106-42-3 | 888.2 | MS, IHL, LRI | 7.52x105 | 1.13x10^5^ | 6.36x10^5^ | 1.61x10^5^ | 2.36x10^5^ | 3.56x10^5^ | 2.25x10^4^ | 1.16x10^4^ | A |
| Ethylbenzene | 100-41-4 | 891.4 | MS | 7.52x10^5^ | 6.53x10^4^ | ND | ND | ND | ND | ND | ND | - |
| o-Xylene | 95-47-6 | 929.7 | MS | 5.28x10^5^ | 2.60x10^4^ | 2.32x10^5^ | 2.61x10^5^ | ND | ND | ND | ND | - |
| o-Cymene | 527-84-4 | 1059.4 | MS | ND | 8.20x10^4^ | 1.94x10^5^ | 2.75x10^5^ | ND | ND | ND | ND | - |
| Phenol | 108-95-2 | 1104.5 | MS, IHL, LRI | 5.95x10^5^ | 1.85x10^6^ | 3.23x10^6^ | 5.08x10^6^ | 4.86x10^6^ | 6.17x10^6^ | 1.00x10^5^ | 8.47x10^4^ | A |
| p-Cresol | 106-44-5 | 1195 | MS, IHL, LRI | 9.80x10^5^ | 1.51x10^6^ | 2.25x10^8^ | 1.93x10^8^ | 2.06x10^8^ | 1.93x10^8^ | 5.82x10^5^ | 2.90x10^5^ | AO |
| 3/4-Ethylphenol | 620-17-7/123-07-9 | 1286 | MS | 1.99x10^5^ | 5.93x10^5^ | ND | ND | ND | ND | ND | ND | O |
| Benzene, 1,3-bis(1,1-dimethylethyl)- | 1014-60-4 | 1287.7 | MS | ND | ND | 8.05x10^4^ | 1.93x10^4^ | 1.30x10^5^ | 8.11x10^4^ | ND | ND | - |
| Benzothiazole | 95-16-9 | 1296.2 | MS, IHL, LRI | 1.34x10^5^ | ND | ND | ND | ND | ND | 7.36x10^4^ | 3.39x10^4^ | O |
| 2-Methoxy-4-vinylphenol | 7786-61-0 | 1411.9 | MS, IHL | 1.01x10^5^ | 1.16x10^7^ | 3.99x10^5^ | 3.14x10^5^ | 1.27x10^6^ | 5.93x10^5^ | 3.63x10^5^ | 4.19x10^4^ | AO |
| Indole | 110-38-3 | 1430.8 | MS, IHL, LRI | 2.72x10^5^ | 7.09x10^5^ | 7.82x10^7^ | 4.20x10^5^ | 7.49x10^7^ | 2.06x10^6^ | 1.49x10^4^ | 1.61x10^4^ | - |
| **Ketones** | | | | | | | | | | | | |
| Acetone | 67-64-1 | 491.9 | MS, IHL, LRI | 9.47x10^5^ | 1.81x10^6^ | 4.67x10^6^ | 2.72x10^6^ | 2.79x10^6^ | 1.87x10^6^ | 2.40x10^5^ | 3.94x10^5^ | A |
| 2,3-Butanedione (Diacetyl) | 431-03-8 | 574.6 | MS, IHL, LRI | 3.03x10^5^ | 1.08x10^6^ | 1.46x10^5^ | 4.79x10^5^ | 4.95x10^5^ | 6.61x10^5^ | 1.12x105 | 1.65x10^5^ | AO |
| 2-Pentanone | 116-09-6 | 704.2 | MS, IHL, LRI | 5.74x10^4^ | 4.55x10^5^ | 1.24x10^5^ | 1.63x10^5^ | 6.37x10^5^ | 2.06x10^5^ | 4.38x10^4^ | 3.61x10^4^ | A |
| 1-Hydroxy-2-propanone | 116-09-6 | 709.8 | MS, IHL | ND | ND | ND | ND | ND | ND | 6.83x10^5^ | 9.41x10^5^ | M |
| Methyl isobutyl ketone | 108-10-1 | 764.5 | MS, IHL | 2.42x10^5^ | ND | ND | ND | 4.82x10^4^ | 4.20x10^4^ | 2.34x10^4^ | 1.34x10^4^ | O |
| 2-Heptanone | 110-43-0 | 931.4 | MS, IHL, LRI | 3.27x10^5^ | 5.94x10^5^ | 9.14x10^5^ | 1.64x10^6^ | 1.53x10^5^ | 3.66x10^5^ | 1.37x10^5^ | 1.97x10^4^ | AO |
| 2-Hexanone | 591-78-6 | 1132.5 | MS, IHL, LRI | 5.45x10^4^ | ND | ND | ND | ND | ND | ND | ND | - |
| Acetophenone | 98-86-2 | 1343.3 | MS, IHL | 5.95x10^5^ | ND | 1.23x10^6^ | ND | 6.80x10^5^ | 1.25x10^5^ | 6.74x10^4^ | 5.18x10^4^ | - |
| 2-Undecanone | 112-12-9 | 1546.2 | MS | ND | ND | 1.71x10^6^ | 3.59x10^6^ | 7.26x10^4^ | ND | 1.02x10^5^ | 7.08x10^4^ | O |
| 2-Tridecanone | 593-08-8 | 1547.6 | MS | ND | ND | ND | ND | ND | ND | 2.60x10^5^ | 1.79x10^5^ | MO |
| 2-Butanone | 78-93-3 | 638.7 | MS | 3.17x10^5^ | 6.64x10^6^ | 6.47x10^5^ | 7.12x10^6^ | 8.59x10^5^ | 1.19x10^7^ | ND | ND | - |
| 1-Hydroxy-2-propanone | 116-09-6 | 733.3 | MS | 2.24x10^6^ | 6.00x10^6^ | 3.43x10^6^ | 3.77x10^6^ | 4.05x10^6^ | 3.65x10^6^ | ND | ND | - |
| 2-Hydroxy-3-pentanone | 5704-20-1 | 867.9 | MS | ND | 4.92x10^6^ | ND | ND | ND | 1.00x105 | ND | ND | - |
| 2-Nonanone | 821-55-6 | 1139.7 | MS | ND | ND | 2.45x10^6^ | 6.25x10^6^ | ND | 1.02x10^6^ | ND | ND | - |
| 2-Pyrrolidinone | 88-12-0 | 1196 | MS | 6.27x10^4^ | 7.44x10^5^ | 8.21x10^4^ | ND | 2.32x10^5^ | ND | 2.28x10^5^ | 9.37x10^4^ | O |
| **Lactones** | | | | | | | | | | | | |
| 2(3H)-Furanone, dihydro-4-hydroxy- | 5469-16-9 | 1382 | MS | ND | ND | ND | ND | ND | ND | 1.43x10^6^ | 1.71x10^6^ | M |
| γ -Butyrolactone | 96-48-0 | 1021.1 | MS, IHL, LRI | 5.08x10^4^ | 1.04x10^6^ | ND | ND | ND | ND | 2.85x10^4^ | 6.91x10^4^ | O |
| 2(5H)-Furanone | 497-23-4 | 1026.3 | MS, LRI | ND | 8.62x10^5^ | ND | ND | ND | ND | 7.72x105 | 1.08x10^6^ | - |
| γ -Hexalactone | 695-06-7 | 1163 | MS, IHL, LRI | 7.49x10^4^ | 3.89x10^5^ | ND | ND | ND | ND | 1.95x10^4^ | 8.67x10^4^ | O |
| γ -Nonalactone | 104-61-0 | 1485 | MS, IHL, LRI | 2.68x10^5^ | 2.16x10^7^ | 1.50x10^7^ | 1.20x10^7^ | 5.81x10^6^ | 6.84x10^6^ | 3.34x10^4^ | 1.84x10^5^ | AO |
| **Pyrazines and Pyradines** | | | | | | | | | | | | |
| Pyrazine | 290-37-9 | 753 | MS | ND | ND | ND | ND | ND | ND | 1.59x10^4^ | 2.86x10^4^ | M |
| Pyridine | 110-86-1 | 789.1 | MS | 1.04x10^4^ | 5.52x10^4^ | 6.43x10^4^ | 1.28x10^5^ | 1.29x10^4^ | 6.90x10^4^ | 6.22x10^3^ | 6.74x10^3^ | A |
| 2,5-Dimethylpyrazine | 123-32-0 | 950 | MS, IHL, LRI | ND | 5.57x10^5^ | 8.52x10^4^ | 1.34x10^5^ | ND | ND | 0.00x00 | 7.27x10^4^ | O |
| 2,3-Dimethylpyrazine | 5910-89-4 | 959 | MS, IHL, LRI | ND | 3.29x10^5^ | ND | ND | ND | ND | 3.77x10^3^ | 5.71x10^4^ | O |
| Pyrazine, trimethyl- | 14667-55-1 | 1044.2 | MS | ND | 5.30x10^5^ | ND | ND | ND | ND | ND | ND | - |
| Pyrazine, 3-ethyl-2,5-dimethyl- | 13360-65-1 | 1115.8 | MS | ND | 1.07x10^5^ | ND | ND | ND | ND | ND | ND | - |
| Pyrazine, tetramethyl- | 1124-11-4 | 1123.9 | MS | ND | 2.55x10^5^ | ND | ND | ND | ND | ND | ND | - |
| **Sulphurs** | | | | | | | | | | | | |
| Methanethiol | 90500-11-1 | 460.1 | MS, IHL, LRI | 1.89x10^4^ | 2.71x10^5^ | 6.16x10^4^ | 2.87x10^5^ | 5.07x10^5^ | 3.46x10^5^ | 4.55x10^5^ | 4.79x10^5^ | O |
| Dimethyl sulfide | 75-18-3 | 538 | MS | 5.40x10^5^ | 7.27x10^4^ | 1.39x10^5^ | 4.59x10^4^ | 9.83x10^6^ | 6.76x10^5^ | ND | ND | - |
| Carbon disulfide | 75-15-0 | 546.6 | MS | ND | 5.75x10^4^ | 1.86x10^5^ | 1.32x10^5^ | 2.34x10^5^ | 1.72x10^5^ | ND | ND | - |
| Disulfide, dimethyl | 624-92-0 | 754.6 | MS | 2.43x10^5^ | 7.52x10^5^ | 9.68x10^5^ | 8.26x10^5^ | 1.44x10^7^ | 3.23x10^6^ | 3.74x10^4^ | 7.44x10^4^ | - |
| Dimethyl sulfone | 67-71-0 | 1056 | MS, IHL, LRI | ND | ND | ND | ND | ND | ND | 2.49x10^3^ | 2.08x10^4^ | M |
| **Other** | | | | | | | | | | | | |
| 1,3-Pentadiene | 1574-41-0 | 534.5 | MS | 2.92x10^5^ | ND | ND | ND | ND | ND | ND | ND | - |
| Methacrolein | 78-85-3 | 615.3 | MS | 1.04x10^4^ | 5.58x10^3^ | ND | ND | ND | ND | ND | ND | - |
| Trichloromethane | 67-66-3 | 655.1 | MS | ND | ND | 6.36x10^3^ | ND | ND | ND | ND | ND | - |
| Mercaptoacetone | 24653-75-6 | 730.3 | MS | ND | ND | ND | ND | 4.18x10^4^ | ND | ND | ND | - |
| Acetoin | 513-86-0 | 777.4 | MS | ND | 2.22x10^7^ | ND | ND | 2.28x10^6^ | 1.09x10^5^ | ND | ND | - |
| Propylene glycol | 57-55-6 | 833.1 | MS | ND | 6.64x10^5^ | ND | ND | ND | ND | ND | ND | - |
| 2,3-Butanediol, [S-(R*,R*)]- | 19132-06-0 | 867.7 | MS | ND | 1.28x10^7^ | ND | 3.08x10^5^ | 7.32x10^4^ | 4.87x10^5^ | ND | ND | - |
| 2,3-Butanediol | 513-85-9 | 870.0 | MS | ND | 1.28x10^7^ | ND | 5.82x10^5^ | 7.32x10^4^ | 5.03x10^5^ | ND | ND | - |
| 1H-Pyrrole, 2-methyl- | 636-41-9 | 918.1 | MS, IHL, LRI | 9.44x10^3^ | 3.71x10^4^ | 3.14x10^4^ | 5.40x10^4^ | 1.70x10^5^ | 6.99x10^4^ | 4.94x10^3^ | 3.94x10^3^ | A |
| 1H-Pyrrole, 3-methyl- | 2703-17-5 | 917.6 | MS | 9.44x10^3^ | 3.71x104 | ND | ND | 1.70x10^5^ | 6.99x10^4^ | ND | ND | - |
| Styrene | 100-42-5 | 929.5 | MS | 3.02x10^5^ | 3.05x10^5^ | 9.50x10^5^ | 4.12x105 | 1.87x10^5^ | 1.97x10^5^ | ND | ND | - |
| Cyclohexanone | 108-94-1 | 962.3 | MS | 7.30x10^4^ | ND | ND | ND | 7.30x10^4^ | ND | ND | ND | - |
| 3-Methyl-2,5-furandione | 110-00-9 | 1050.8 | MS | ND | ND | ND | ND | ND | ND | 4.96x10^5^ | 8.05x10^5^ | M |
| 5-Hepten-2-one, 6-methyl- | 110-93-0 | 1036.3 | MS | 7.79x10^5^ | 4.21x10^5^ | 1.55x10^6^ | 3.32x10^6^ | 1.77x10^5^ | 7.15x10^5^ | ND | ND | - |
| 2-Furancarboxaldehyde, 5-methyl- | 620-02-0 | 1040.7 | MS | 6.33x10^4^ | ND | ND | ND | ND | ND | ND | ND | - |
| 3-Carene | 13466-78-9 | 1051.4 | MS | ND | ND | 4.50x10^4^ | ND | ND | ND | ND | ND | - |
| 1H-Pyrrole-2,5-dione | 541-59-3 | 1102.9 | MS | ND | ND | ND | ND | ND | ND | 8.09x10^3^ | 9.32x10^3^ | M |
| Linalool | 78-70-6 | 1145.6 | MS | 1.69x10^6^ | 8.69x10^6^ | ND | ND | ND | ND | ND | ND | - |
| Maltol | 88-12-0 | 1193 | MS, LRI | ND | ND | ND | ND | ND | ND | 1.28x10^6^ | 2.39x10^6^ | MO |
| Damascenone | 23696-85-7 | 1467.5 | MS | ND | 1.06x10^6^ | ND | 3.33x10^5^ | ND | ND | ND | ND | - |

A = compound identified in all sample types; M = compound identified in milk only; O = compound is odour active; ND = not detected.

Levels of volatile compounds are expressed as abundances (mean values from 3 extractions from each sample.

LRI: retention index on a DB-624 UI column; IM: identification method; MS: spectra comparison using NIST mass spectral database; IHL: in-house library created using authentic compounds with target and qualifier ions and linear RI for each compound; LRI: RI agree with literature values.
